# AaMYC3 bridges the regulation of glandular trichome density and artemisinin biosynthesis in *Artemisia annua*

**DOI:** 10.1111/pbi.14449

**Published:** 2024-08-27

**Authors:** Mingyuan Yuan, Yinguo Sheng, Jingjing Bao, Wenkai Wu, Guibin Nie, Lingjian Wang, Junfeng Cao

**Affiliations:** ^1^ State Key Laboratory of Pharmaceutical Biotechnology School of Life Sciences, Nanjing University Nanjing 210023 China; ^2^ National Key Laboratory of Plant Molecular Genetics CAS Center for Excellence in Molecular Plant Sciences/Shanghai Institute of Plant Physiology and Ecology, Chinese Academy of Sciences Shanghai 200032 China; ^3^ School of Life Sciences, Centre for Cell & Developmental Biology, State Key Laboratory of Agrobiotechnology The Chinese University of Hong Kong Shatin Hong Kong

**Keywords:** Artemisinin, *Artemisia annua*, AaMYC3, gland‐secreting trichome (GST), jasmonic acid (JA)

## Abstract

Artemisinin, the well‐known natural product for treating malaria, is biosynthesised and stored in the glandular‐secreting trichomes (GSTs) of *Artemisia annua.* While numerous efforts have clarified artemisinin metabolism and regulation, the molecular association between artemisinin biosynthesis and GST development remains elusive. Here, we identified AaMYC3, a bHLH transcription factor of *A. annua*, induced by jasmonic acid (JA), which simultaneously regulates GST density and artemisinin biosynthesis. Overexpressing *AaMYC3* led to a substantial increase in GST density and artemisinin accumulation. Conversely, in the RNAi‐*AaMYC3* lines, both GST density and artemisinin content were markedly reduced. Through RNA‐seq and analyses conducted both *in vivo* and *in vitro*, AaMYC3 not only directly activates *AaHD1* transcription, initiating GST development, but also up‐regulates the expression of artemisinin biosynthetic genes, including *CYP71AV1* and *ALDH1*, thereby promoting artemisinin production. Furthermore, AaMYC3 acts as a co‐activator, interacting with AabHLH1 and AabHLH113, to trigger the transcription of two crucial enzymes in the artemisinin biosynthesis pathway, ADS and DBR2, ultimately boosting yield. Our findings highlight a critical connection between GST initiation and artemisinin biosynthesis in *A. annua*, providing a new target for molecular design breeding of traditional Chinese medicine.

## Introduction

Glandular trichomes play an important role in plants, serving as vital organs for defending against external stresses and facilitating the biosynthesis of various secondary metabolites (Feng *et al*., 2021; Handley *et al*., 2005; Schilmiller *et al*., 2008; Schuurink and Tissier, 2020). For example, in tomato glandular trichomes, a variety of terpenes are biosynthesised to defend against pathogens and herbivores (Huang and Osbourn, 2019; Yang *et al*., 2021); Cotton defends itself against pests by regulating the accumulation of gland gossypol (Gao *et al*., 2020; Lin *et al*., 2023); and alkane metabolism in tobacco glandular trichomes affects the cold resistance (Yu *et al*., 2022). Notably, in medicinal plants, glands play a prominent role in secondary metabolic biosynthesis (Tang *et al*., 2022). For example, *Tussilago Farfara* synthesizes and stores polyphenols and flavonoids in its glandular trichomes (Muravnik *et al*., 2016). Other renowned instances of valuable natural products sourced from glandular secretory trichomes (GSTs) encompass the biosynthesis of alkaloids in *Withania somnifera* L. (Munien *et al*., 2015), and artemisinin in *A. annua* (Cao *et al*., 2023; Yan *et al*., 2017, 2018).

The molecular mechanisms underlying both trichome development and secondary metabolic biosynthesis in plants form an inseparable unity (McConkey *et al*., 2000). The GL1‐GL3/EGL3‐TTG1 (MBW) complex model, which activates the downstream HD‐Zip IV gene *GL2* to promote trichome growth, may represent a general mechanism for trichome development (Cui *et al*., 2022; Wang *et al*., 2019), particularly for non‐secretory glandular trichomes (NGTs). Despite variations observed among GSTs, the HD‐zip genes retain their central role in GST development in horticulture crops like tomatoes (Hua *et al*., 2021; Wu *et al*., 2024a) and *A. annua* (Xie *et al*., 2021b). NGTs provide mechanical protection and defence for plants against various stresses (Karabourniotis *et al*., 2020; Kaur and Kariyat, 2020), whereas GSTs predominantly serve as secondary metabolic sites in most plants, especially for terpenoids biosynthesis (Wang *et al*., 2008). Terpenes secreted by hop GSTs impart flavour and aroma to beer, while lavender GSTs produce volatile terpenes (Wang *et al*., 2008; Zhang *et al*., 2023). Volatile terpenoids are closely associated with glandular trichome development in tomato (Xu *et al*., 2018). SlEOT1, a TF specifically expressed in GSTs, directly regulates TPS5 to promote terpene biosynthesis in tomato (Spyropoulou *et al*., 2014). JA‐induced SlMYC1 and SlMYC2 play dual roles in stress‐induced specialized metabolism in *Solanum lycopersicum* (Swinnen *et al*., 2022). Furthermore, SlMYC1 positively regulates glandular trichome formation and terpene biosynthesis, serving as a key integrator of trichome development and terpene metabolism (Xu *et al*., 2018). Additionally, cotton GoPGF regulates the initiation of pigment glands while directly activating downstream targets to regulate cotton phenol biosynthesis (Ma *et al*., 2016).

Artemisinin, a sesquiterpene lactone, is synthesized in the GSTs of *A. annua* and widely utilized for malaria treatment. The GST cells consist of 10 composite cells (Olofsson *et al*., 2012), continuously synthesizing and storing artemisinin during development. It has been shown that *AaHD1* is the core factor controlling the density of GST, which is conserved between NGTs and GSTs (Hassani *et al*., 2020; Wang *et al*., 2019). The differences are that AaMYB16 and AaMYB5 interact with AaHD1 to, respectively, promote and inhibit GST density (Xie *et al*., 2021b). Moreover, *AaGSW2* and *AaTAR2*, are identified as the essential downstream target gene of AaHD1 with a great impact on GST initiation (Xie *et al*., 2021a). The expression of these genes is mainly concentrated during the young leaf period and decreases as the leaf develops and matures, which is consistent with the initiation and density of GST (Xie *et al*., 2021a). However, these genes do not participate in artemisinin biosynthesis directly, as transcription factors (TFs) specifically expressed in GSTs regulate the artemisinin biosynthesis, such as AabHLH1 and AabHLH113 (Ji *et al*., 2014; Yuan *et al*., 2023). Although the mechanisms of glandular trichome development and artemisinin biosynthesis in *A. annua* have been extensively studied, their relationship remains unclear, indicating a gap in understanding the regulation network between the two.

Here, we identified a new TF, AaMYC3, which enhances the expression of *AaHD1* binding to and activating the promoter of *AaHD1*, thereby promoting GST density. Meanwhile, AaMYC3 boosts the artemisinin biosynthesis‐specific route as a TF for *CYP71AV1* and *ALDH1*, while interacting with AabHLH1 and AabHLH113 to function as a co‐activator to transcribe *ADS* and *DBR2*. The findings reveal the genetic relationship between GSTs development and artemisinin biosynthesis, which could be a potential candidate for the improvement of *A. annua*.

## Materials and methods

### Plant materials and treatments

The *A. annua* ‘Huhao1’ was used as the wild type and the transgenic receptor. Tobacco plants were sourced from the long‐term preservation of *Nicotiana benthamiana* (Zhao *et al*., 2018). Sterilized *A. annua* seeds were sown in MS medium (Sigma‐Aldrich, USA) supplemented with 30% sucrose and 10% agar. Both tobacco and transgenic *A. annua* were cultivated in a mixture of perlite, vermiculite, and pine bark (6:3:1). *A. annua* seedlings in MS medium served as controls for transgenic experiments, while tobacco plants were used for transient transformation. All plants were grown under a 16‐h light/8‐h dark cycle at 28 °C.

### Immunoprecipitation‐mass spectrometry

Immunoprecipitation‐Mass Spectrometry (IP‐MS) assays were conducted using IP buffer (50 mM Tris–HCl, 150 mM NaCl, 15% glycerol, 0.5% NP‐40) and plant protein extraction buffer (50 mM Tris–HCl, 300 mM NaCl, 1 mM DTT, 1% TritoX‐100, 1xPIC). The 9th true leaves (100 mg) of OE‐*AabHLH113* and OE‐*AaMYC3* were homogenized in 500 μL IP buffer, incubated on ice for 15 min, and centrifuged to collect the supernatant. Flag antibody‐bound magnetic beads were incubated with the supernatant at 4 °C for 3 h, washed thrice with IP buffer, and subjected to electrophoresis (Chi‐Biotech, Shenzhen, China). Peptides were solubilized in 0.1% formic acid and analysed via Orbitrap Fusion Lumos connected to an EASY‐nanoLC 1200 system (Thermo Fisher Scientific, MA, USA). A 5 μL peptide sample was chromatographed on a 25 cm column (75 μm ID, 1.9 μm resin) using a 60‐min gradient from 4% to 95% buffer B (80% ACN, 0.1% FA), with a flow rate of 300 nL/min at 40 °C. Mass spectra were acquired at 2 kV and analysed with PEAKS Studio v10.6 against the *A. annua* database (v.2024, UniProt). MS‐identified proteins were listed in Tables S1 and S2.

### MeJA treatment and tissue expression analysis

Thirty‐day‐old *A. annua* seedlings were used for expression analysis. The MeJA treatment followed the protocol as described previously (Yuan *et al*., 2023), involving the collection of leaves from the same leaf age in both the *A. annua* treatment and control groups at various time points (0, 3, 6, 12, 24, 48 h, 0, 1, 3, 5, and 7 days). Tissue expression analysis and leaf age expression analysis were conducted, utilizing different tissues from *A. annua*, including buds, flowers, leaves, and stems, as well as leaves of varying ages (Ma *et al*., 2018).

Total RNA was isolated from the samples using the RNAprep Pure Plant Kit (DP441, Tiangen Biotech, Beijing, China) (Wang *et al*., 2021). For cDNA synthesis, 1 μg of the extracted RNA served as a template in reactions with the TransScript® II First‐Strand cDNA Synthesis SuperMix (AH301‐03, TransGen Biotech, Beijing, China) (Shi *et al*., 2022). Subsequent quantitative real‐time PCR (qRT‐PCR) analyses employed a 10‐fold dilution of the synthesized cDNA products, utilizing SYBR® Green Pro Taq HS (AG11701, Accurate Biotechnology (HUNAN) CO. LTD, ChangSha, China) for detection with primers in Table S3.

### RNA‐seq

RNA sequencing was performed on leaves (leaf 0 to leaf 9) from uniformly grown transgenic and WT seedlings at Chi‐Biotech, Shenzhen, China. Clean reads were obtained by discarding those containing adapters, poly‐N, and low‐quality sequences (Jiao *et al*., 2023). The refined data were analysed to ascertain Q20, Q30, GC‐content, and sequence duplication rates (Shi *et al*., 2023). All further analyses were conducted on this high‐quality data set. Gene annotation was achieved by aligning with the published *A. annua* genome (Shen *et al*., 2018), and functional categorization was done using the KEGG pathways database. Gene expression levels were normalized and quantified as RPKM. Differentially expressed genes were identified with an FDR ≤0.001 and an absolute log2 fold change ≥1, using a statistical random test (*P* < 0.05) (Cao *et al*., 2020b).

### Co‐expression analysis

Previously we have analysed the expression mode of the bHLH family of genes in *A. annua* (Yuan *et al*., 2023). We noted 88 AabHLH transcription factors (TFs) expressed at high levels in *A. annua* (Chang *et al*., 2023). To screen for TFs associated with artemisinin gland‐secreting trichomes (GSTs) and artemisinin biosynthesis, TBtools was used to co‐expression analysis of these 88 *AabHLH*s to the artemisinin biosynthetic genes *ADS*, *CYP71AV1*, *DBR2*, *DBR2*‐*Like*, and *ALDH1*, in the young leaves (YL, SRR6472941), buds (SRR6472946), flowers (SRR6472948), stem (SRR6472943), old leaf (OL, SRR6472942), bud trichomes (BT, SRR019548), epidermis (SRR6472945) and mature leaf trichomes (MLT, SRR019547). Transcriptome data was obtained from NCBI (PRJNA416223) (Shen *et al*., 2018). S_ALMON_ (v.0.13.1) (Patro *et al*., 2017) quantifies transcript abundance of artemisinin biosynthetic genes and *AabHLH*s, and TBtools (v.0.665.0) (Chen *et al*., 2023; Zhang *et al*., 2021) was used for hierarchical clustering analysis.

### Phylogenetic tree analysis

To construct the phylogenetic tree of Maximum likelihood (ML) (Cao *et al*., 2020a; Huang *et al*., 2022), we obtained the candidate amino acid sequences of 8 AabHLHs TF from *A. annua* genome and the amino acid sequences of all AtbHLH members of *Arabidopsis thaliana* from TAIR (https://www.arabidopsis.org). Muscle5.1 (Edgar, 2022) was used to carry out the sequence comparison, and subsequently, the compared sequences were trimmed by the trimAl., finally IQ‐TREE (v2.2.0; http://www.iqtree.org) was used to construct the ML Phylogenetic tree (Cao *et al*., 2022).

### Transformation of *A. annua*

*AaMYC3* was inserted into the pHB‐Flag plasmid, driven by the double CaMV35S promoter. A 357‐bp fragment of the *AaMYC3* coding sequence was inserted into the pHANNIBLE plasmid, and then the RNAi region obtained was constructed into the pBIN19 plasmid. These reconstructed plasmids were introduced into the *Agrobacterium tumefaciens* strain EHA105 and individually used to transform *A. annua* as described previously (Yuan *et al*., 2023). All primers are listed in Table S3.

### Subcellular localization

To investigate the subcellular localization of AaMYC3, the coding sequence of *AaMYC3* was inserted into the pCAMBIA1300‐GFP plasmid, using the empty plasmid pCAMBIA1300‐GFP as the control. Subsequently, subcellular localization assays were performed as described previously (Yuan *et al*., 2023). All primers are listed in Table S3.

### GUS histochemical staining

Specific primers were designed and the 1023‐bp region of the *AaMYC3* promoter was obtained from the *A. annua* genomic DNA library. pAaMYC3 was inserted into the pCAMBIA1305.1‐GUS plasmid. Subsequently, an engineered *A. tumefaciens* strain EHA105 containing the recombinant plasmid was obtained and transformed *A. annua* as described previously (Yuan *et al*., 2023). Histochemical staining assays for GUS activity in the obtained transgenic *A. annua* were performed as described previously (Yuan *et al*., 2023). All primers are listed in Table S3.

### Glandular trichome density counting

Mature leaves of *A. annua* plants (leaf 9) were collected to count the density of glandular trichomes. Images of each leaf adaxial side were obtained using a fluorescence microscope (Leica DM2500, Leica, Germany) under excitation at 460 nm. Leaf area was measured and the number of glandular trichomes was counted using the ImageJ (http://rsb.info.nih.gov/ij) as described previously (Yan *et al*., 2017). Three biological replicates were performed for all lines.

### Dual‐luciferase assays

The promoters of artemisinin biosynthetic genes (*ADS*, *CYP71AV1*, *DBR2* and *ALDH1*) and *AaHD1* were inserted into pGreenII0800‐LUC plasmid as reporters, respectively. The coding sequences of *AaMYC3*, *AabHLH1* and *AabHLH113* were inserted into the pHB‐YFP plasmid as effectors, respectively. The pHB‐YFP plasmid was used as a negative control. Subsequently, dual‐luciferase assays (Dual‐LUC) were performed as in the previous method (Shangguan *et al*., 2021). The Dual‐LUC experiments were all performed in 3 biological replicates. All primers are listed in Table S3.

### Luciferase luminescence detection assays

Transient transformation in tobacco followed the protocol established for Dual‐LUC assays (Cao *et al*., 2023). Tobacco leaves were infiltrated with 0.5 mM D‐Luciferin Potassium Salt (Beyotime Biotechnology, Shanghai, China), consistent with the methodology outlined previously (Yuan *et al*., 2023). LUC luminescence was subsequently visualized using a Tanon‐5200 live plant imaging system (Tanon, Shanghai, China).

### Bimolecular luciferase complementation assays

For bimolecular luciferase complementation (BiLC) assays, the coding sequence of *AaMYC3* was inserted into the pJW771‐LUCn plasmid, while the coding sequences of *AabHLH113* and *AabHLH1* were inserted into the pJW772‐LUCc plasmid, respectively. Transient transformation of tobacco was subsequently performed as previously described (Cao *et al*., 2023). Luciferase luminescence imaging was performed using a living plant imaging system and 0.5 mM _D_‐luciferin potassium salt as a luminescent substrate (Cao *et al*., 2023). All primers are listed in Table S3.

### CUT&tag‐qPCR assays

For CUT&Tag‐qPCR assays, nuclei from 100 mg of fresh OE‐*AaMYC3* leaves were isolated. The Hyperactive Universal CUT&Tag Assay Kit (Vazyme, Nanjing, China) was employed, utilizing a mouse anti‐Flag antibody (1:20 000, Sigma‐Aldrich, Shanghai, China) as the primary antibody, and a goat anti‐mouse secondary antibody (1:100, Vazyme, Nanjing, China). Normal mouse IgG (1:20 000, Sigma‐Aldrich, Shanghai, China) served as the control. Post pA/G‐Tnp treatment for fragmentation, the DNA library was extracted, amplified, purified, and analysed via RT‐qPCR. Primer sequences are detailed in Table S4.

### Yeast one‐hybrid assays

The coding sequence of *AaMYC3* was inserted into the pB42AD plasmid as prey. Meanwhile, *AaHD1* promoter and artemisinin biosynthetic gene promoters truncated to different fragment lengths, were individually inserted into pLacZ plasmid as baits. Subsequently, the yeast one‐hybrid (Y1H) assays were performed as described previously (Yuan *et al*., 2023). All primers and sequences are listed in Table S5, respectively.

### Yeast two‐hybrid assays

To perform yeast two‐hybrid (Y2H) assays, *AabHLH1* and *AabHLH113* coding sequences were individually inserted into the pGBKT7 plasmid (baits), while the coding sequence of *AaMYC3* was inserted into the pGADT7 plasmid (prey). Subsequently, yeast transformation assays were performed and the interaction of AaMYC3 with AabHLH1 and AabHLH113 was verified using different defective media, respectively, as described previously (Zhang *et al*., 2018). All primers are listed in Table S6.

### Measurement of metabolite by HPLC

Three‐month‐old transgenic, wild‐type, and JA‐treated wild‐type *A. annua* leaves were individually collected and dried at 40 °C. Subsequently, 0.2 g dried leaves were weighed to extract artemisinin and dihydroartemisinic acid, where the extraction method was as previously described (Yuan *et al*., 2023). Artemisinin and dihydroartemisinic acid were detected using high‐performance liquid chromatography (HPLC) as previously described (Yuan *et al*., 2023). At least three biological replicates were performed during extraction and detection.

### Co‐immunoprecipitation assays

To perform co‐immunoprecipitation (Co‐IP) assays, the coding sequence of *AaMYC3* was inserted into pCAMBIA1300‐YFP, while *AabHLH1* and *AabHLH113* were individually inserted into pCAMBIA2301, where pCAMBIA1300‐YFP served as a negative control. Co‐IP assays were performed using an Immunoprecipitation Kit (Beyotime Biotechnology, Shanghai, China). Finally, immunoblot analysis was performed using a Western Blot kit (Beyotime Biotechnology, Shanghai, China), in which antibodies were used with anti‐YFP and anti‐Flag monoclonal antibodies produced in mouse (1:10 000 dilution, Sigma‐Aldrich, USA) and HRP‐coupled rabbit anti‐mouse antibody (1:5000 dilution, Sigma‐Aldrich, USA), respectively. All primers are listed in Table S5.

### Electrophoretic mobility shift assays

To perform electrophoretic mobility shift assays (EMSAs), the *AaMYC3* coding sequence was inserted into the pMAL‐c5X plasmid, and MBP‐AaMYC3 recombinant protein was used to obtain using Frdbio® MBP Tagged Protein Purification Kit (Frdbio Bioscience & Technology, Wuhan, China). The recombinant proteins His‐AabHLH1 and MBP‐AabHLH113 were obtained as described previously (Yuan *et al*., 2023). Subsequently, biotin‐tagged and no‐tagged fragments (E‐box and G‐box) were synthesized as probes and competitive probes, respectively (Biosune, Shanghai, China). Finally, EMSA assays were executed using a Chemiluminescent EMSA Kit (Beyotime Biotechnology, Shanghai, China). All probe sequences are listed in Table S7.

## Results

### AaMYC3 associated with GST distribution has a similar expression pattern to artemisinin biosynthetic genes

**Figure 1:**
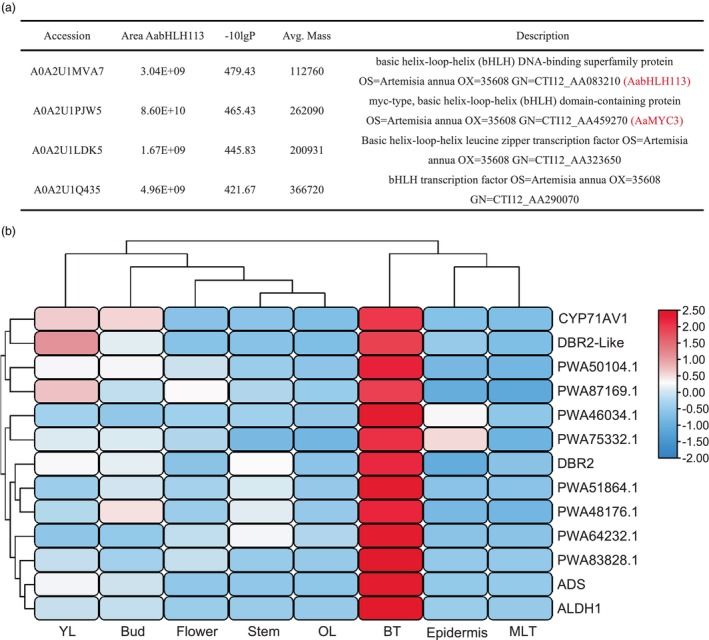
IP‐MS screening of proteins interacting with AabHLH113 in OE‐*AabHLH113* transgenic *Artemisia annua* and Co‐expression analysis of bHLH transcription factors (TFs) in *Artemisia annua*. (a) Proteins with the highest abundance sequenced by IP‐MS. The sequenced peptides in Figure S1. (b) The co‐expression analysis of 88 *AabHLH*s to the artemisinin biosynthetic genes *ADS*, *CYP71AV1*, *DBR2*, *DBR2‐Like*, and *ALDH1*, in the young leaves (YL), buds, flowers, stem, old leaf (OL), bud trichomes (BT), epidermis and mature leaf trichomes (MLT). Eight *AabHLH*s with similar expression patterns to artemisinin biosynthetic genes are presented here. The colour represented the relative expression level from low (blue) to high (red). The entire heatmap is provided in Figure S2.

GSTs densely cover the young buds and leaves of *A. annua*, where sesquiterpenoids, such as artemisinin, accumulate (Xie *et al*., 2021b; Yan *et al*., 2017). GST initiation and density decrease as leaves mature, and genes specifically expressed in GST, such as AaHD1 and AaTAR2, are highly expressed in young leaves, indicating their crucial role in GST initiation (Yan *et al*., 2017; Zhou *et al*., 2020). AabHLH113 has been identified as a crucial regulator within glandular trichomes, steering the synthesis of artemisinin (Yuan *et al*., 2023). To uncover potential regulatory genes associated with artemisinin or GSTs, IP‐MS was conducted with AabHLH113. The screening revealed 24 proteins interacting with AabHLH113, particularly three bHLH proteins with notable peptide enrichment (Figures 1a and S1; Table S1). To delve deeper into the molecular intricacies of artemisinin production and trichome formation, a comprehensive survey identified and detailed bHLH transcription factors across the *A. annua* genome (Chang *et al*., 2023). A particular cluster of 88 AabHLH genes, distinguished by their elevated expression in various tissues, drew our focus (Figure S2). These genes were then subjected to co‐expression analysis alongside artemisinin biosynthetic genes (Figure S2). Notably, eight *AabHLH*s mirrored the expression profiles of *ADS*, *CYP71AV1*, *DBR2*, *DBR2*‐*Like*, and *ALDH1*, predominantly in bud trichomes (BT) (Figure 1b). Merging IP‐MS outcomes with co‐expression data pinpointed PWA51864.1 (CTI12_AA459270) as a gene meriting further exploration. Phylogenetic analysis places PWA51864.1 alongside eight AabHLHs from *A. annua* and AtMYC2, 3, and 4 from *Arabidopsis*, differentiating it from AaMYC2 and introducing it as the new transcription factor AaMYC3 (Figure S3). Given the significant influence of the analogous AtMYCs on the jasmonate (JA) signalling pathway (Kazan and Manners, 2013; Tsuda and Somssich, 2015), *AaMYC3* is likely to be instrumental in the JA‐mediated regulation of GST development and artemisinin biosynthesis in *A. annua*.

**Figure 2:**
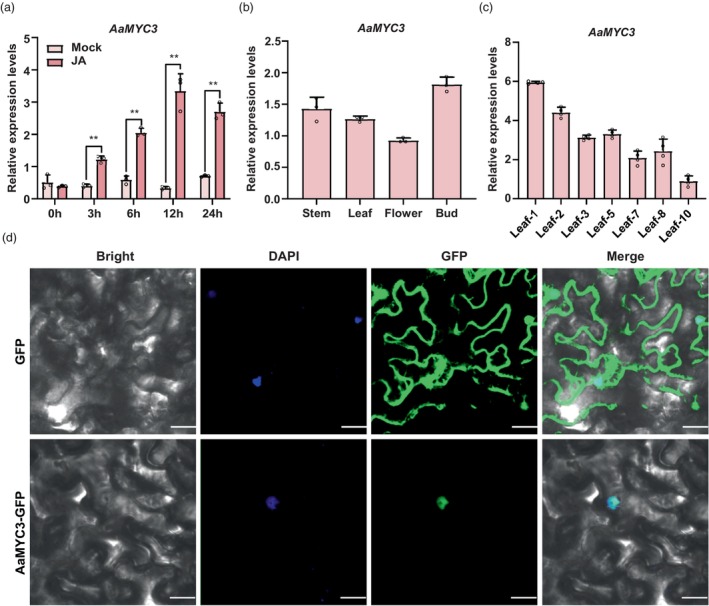
The JA‐induced nuclear regulator AaMYC3 is consistent with the expression pattern of gland‐secreting trichomes (GSTs). (a) Relative expression levels of *AaMYC3* after methyl jasmonate (MeJA) treatment. All data represent the means ± SDs (*n* = 3; **P < 0.01; Student's *t*‐test). (b) Relative expression levels of AaMYC3 in wild‐type *Artemisia annua* plants various tissues (buds, stems, leaves, and flowers). All data represent the means ± SDs (*n* = 3). (c) Relative expression levels of AaMYC3 in wild‐type *Artemisia annua* plants various leaf sequences. All data represent the means ± SDs (*n* = 4). (d) Subcellular localization of AaMYC3. Images show green fluorescent protein (GFP) fluorescence, bright light, 4′,6‐diamino‐2‐phenyllindol (DAPI) staining fluorescence, and in combination in tobacco leaves. Bars:10 μm.

We then examined the relative expression levels of *AaMYC3* after MeJA treatment and observed strong induction of *AaMYC3* (Figure 2a). The transcript levels of *AaMYC3* in different tissues were consistent with the co‐expression analyses through RT‐qPCR, indicating predominant expression of *AaMYC3* in young buds (Figure 2b). GST density decreased with leaf age in *A. annua*. Therefore, we detected the expression pattern of *AaMYC3* in leaves of different ages. *AaMYC3* had a higher transcript level in young leaves with expression levels decreasing as leaf senescence (Figure 2c). Subcellular localization assays support the nuclear localization of AaMYC3 (Figure 2d), consistent with its function as a TF. Subsequently, transgenic *A. annua* plants were produced with pAaMYC3 driving the *GUS* reporter gene. Histochemical analysis localized the signal in the GSTs (Figure S4). Collectively, these data suggest that AaMYC3 likely regulates the development of GSTs and artemisinin biosynthesis.

### Altering AaMYC3 expressions affected GST density and artemisinin production

**Figure 3:**
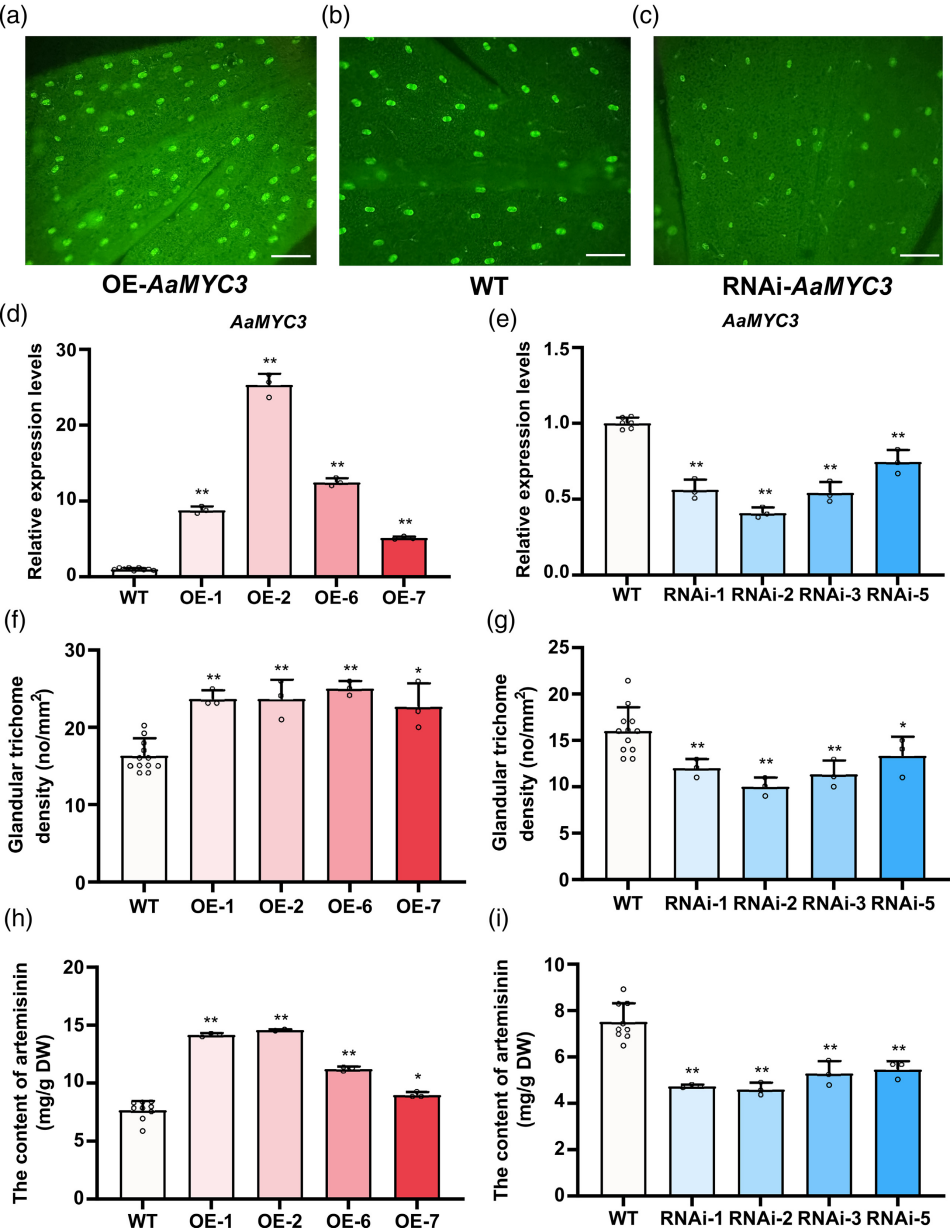
*AaMYC3* expression affects glandular trichome density and artemisinin content. (a–c) Imaging of glandular trichomes on the adaxial side of mature leaves of RNAi‐*AaMYC3* (RNAi‐2), wild‐type (WT), and OE‐*AaMYC3* (OE‐2) *Artemisia annua* plants. Green fluorescent glandular trichomes were captured by the yellow autofluorescence of the glandular trichomes and the blue autofluorescence of the Chl fluorescence microscope. Bars: 200 μm. (d, e) Relative expression levels of *AaMYC3* in OE‐*AaMYC3* and RNAi‐*AaMYC3* lines, compared with wild‐type, respectively. (f, g) Density of glandular trichome on mature leaves on the adaxial side of RNAi‐*AaMYC3*, WT, and OE‐*AaMYC3 A. annua* plants. (h, i) Contents of artemisinin in OE‐*AaMYC3*, WT and RNAi‐*AaMYC3* lines. All data represent the means ±SDs (*n* ≥ 3 as indicated in the figure; *P < 0.05; **P < 0.01; Student's *t*‐test).

To clarify the function of *AaMYC3* in *A. annua*, we generated transgenic lines overexpressing *AaMYC3* (OE‐*AaMYC3*) (Figure S5) and lines with RNA interference of *AaMYC3* (RNAi‐*AaMYC3*) (Figure S6). Observations of mature leaf (leaf 9) adaxial side under fluorescence microscopy revealed a marked contrast in glandular trichome density between the transgenic and wild‐type specimens (Figure 3a‐c, S7). RT‐qPCR analysis showed a substantial elevation of *AaMYC3* transcripts in OE‐*AaMYC3* lines (Figure 3d), with a corresponding decrease in RNAi‐*AaMYC3* lines (Figure 3e), indicating fold changes of 5.14–25.30 and a reduction of 41% to 75%, respectively, against the wild type. Notably, the GST density on the adaxial side of mature leaves in OE‐*AaMYC3* lines was significantly higher, at 1.39–1.53 times that of the control (Figure 3f), while RNAi‐*AaMYC3* lines exhibited a decrease of 17%–38% (Figure 3g). Correspondingly, artemisinin levels were markedly upregulated in OE‐*AaMYC3* lines by 1.43–1.81 times (Figure 3h) and downregulated in RNAi‐*AaMYC3* lines by 27% to 39% (Figure 3i). These results suggest that AaMYC3 is a positive regulator of GST density in *A. annua*.

### AaMYC3 regulates GST density through *AaHD1*

**Figure 4:**
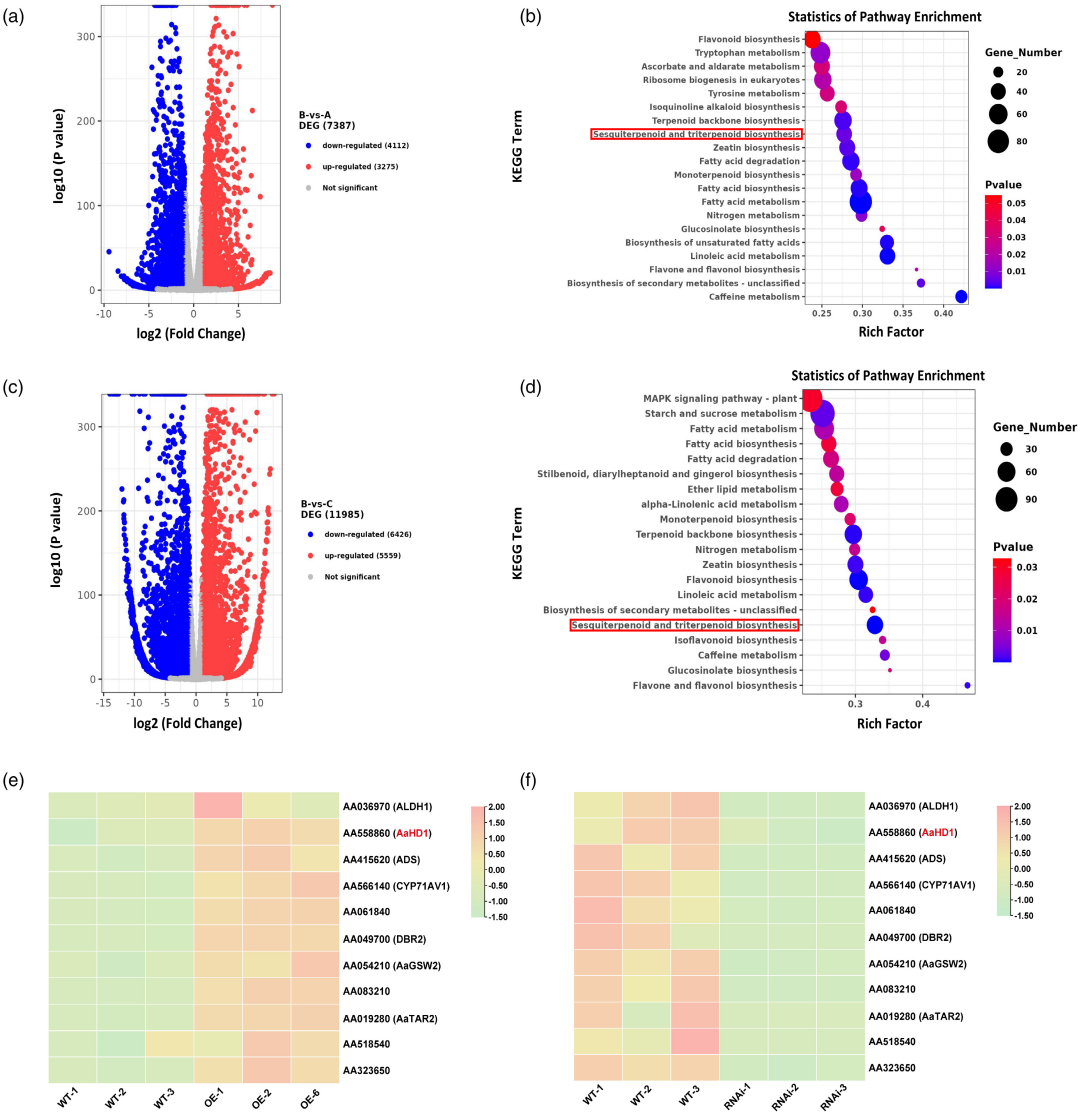
AaMYC3 regulates the expression level of GST and artemisinin biosynthetic genes in RNA‐seq. (a‐b) Volcano graphs of all genes significantly differentially expressed in OE (A), RNAi (C) and WT (B). The screening threshold for differential genes in the analysis was set at *P* < 0.05 and |log2(FoldChange)| > 1. (c‐d) KEGG enrichment analysis in GSEA was performed on the screened differential genes of WT‐vs‐OE. The screening threshold for differential genes in the analysis was set at *P* < 0.05 and |log2(FoldChange)| > 1. (c) and RNAi‐vs‐WT (d) to identify the most prominent biochemical metabolic pathways and signalling pathways in which the differentially expressed genes are involved. (e, f) The heatmap of GST and artemisinin biosynthetic genes, which were significantly up‐regulated in OE compared to WT (e), while the expression levels were significantly down‐regulated in RNAi lines.

**Figure 5:**
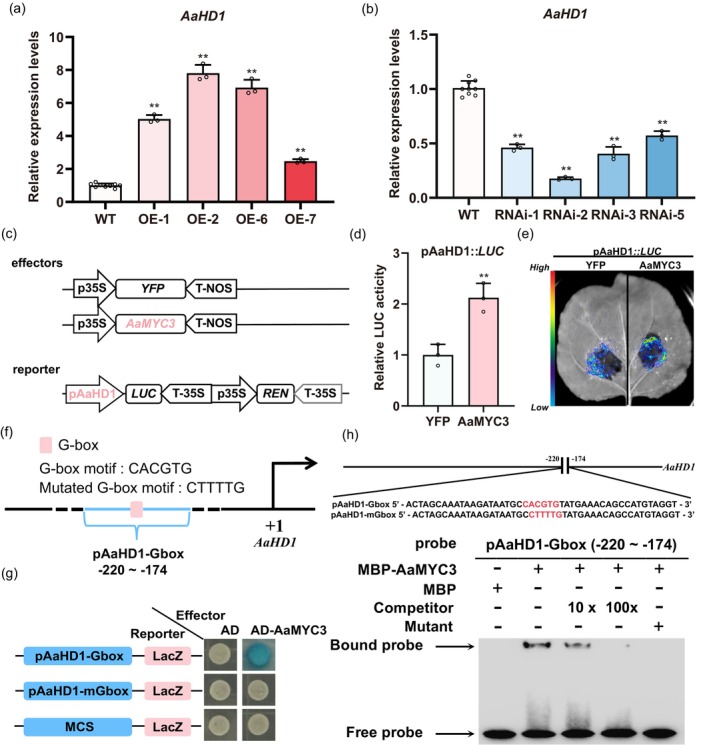
AaMYC3 binds to and activates the G‐box *cis*‐element on the *AaHD1* promoter. (a, b) Relative expression levels of *AaHD1* in OE‐*AaMYC3* and RNAi‐*AaMYC3* lines compared with wild‐type (WT) *A. annua*, respectively. (c) Structural diagrams of effectors (35S::*YFP* and 35S::*AaMYC3*) and reporters (pAaHD1::*LUC*) in dual‐luciferase (Dual‐LUC) assays (REN, renilla luciferase; LUC, firefly luciferase). (d) The activity of *AaHD1* promoters fused to the LUC reporter was determined by Dual‐LUC assays. Relative LUC activity was normalized to the reference REN. Yellow fluorescent protein (YFP) effector was used as a negative control, and the LUC: REN ratios of YFP were set as 1. (e) LUC chemiluminescence imaging system photographed fluorescence in Dual‐LUC assays. (f) Schematic diagrams of the position of the fragment on the promoter of *AaHD1* used in yeast one‐hybrid (Y1H) assays. The positions of potential response G‐box *cis*‐elements are shown as rounded square boxes, and they are numbered according to their distance from the translation initiation site (ATG), which is set as +1. (g) Y1H assays between AaMYC3 and pAaHD1‐Gbox, of which the G‐box (CACGTG) motif was mutated to the mG‐box (CTTTTG) motifs. The data represent five independent experiments, and representative results are shown. (h) Electrophoretic mobility shift assays showed the binding of MBP‐AaMYC3 to the G‐box of the AaHD1 promoter. MBP protein was used as a negative control. Unlabeled pAaHD1‐Gbox at 1×, 10×, and 50× molar ratios as competitors. The G‐box is highlighted in red. All data represent the means ±SDs (*n* ≥ 3 as indicated in the figure; **P < 0.01; Student's *t*‐test). [Correction added on 13 September 2024, after first online publication: the Figures 5a and 8i are revised in this version.]

To decipher the role of *AaMYC3* in regulating GSTs or artemisinin, RNA‐seq was performed with seedlings of OE‐*AaMYC3*, RNAi‐*AaMYC3*, and wild‐type. Principal Component Analysis (PCA) distinctly categorized the samples into three groups (Figure S8). Differential gene expression in OE‐*AaMYC3* and RNAi‐*AaMYC3* lines, relative to wild‐type, was visualized through volcano plots and Venn diagrams (Figures 4a–d and S9a). Notably, the top 20 KEGG pathways enriched included sesquiterpenoid biosynthesis (Figure 4a–d). The sesquiterpene biosynthesis pathway, with the lowest *P*‐value, was highlighted by mapping the Enrichment Score (ES) (Figure S9b,c). Heatmaps of core genes revealed significant expression differences in glandular trichome initiation genes—*AaHD1*, *AaGSW2*, and *AaTAR2*—alongside genes on the artemisinin biosynthesis pathway (*ADS*, *CYP71AV1*, *DBR2*, *ALDH1*) (Figure 4e,f). Given that AaGSW2 and AaTAR2 are downstream of AaHD1 (Schweizer *et al*., 2013; Swinnen *et al*., 2022), we assessed *AaHD1* expression level in transgenic lines, finding a 2.47‐ to 7.80‐fold increase in OE‐*AaMYC3* lines (Figure 5a) and a 43%–83% decrease in RNAi‐*AaMYC3* lines (Figure 5b). In addition *AaHD1*, *AaGSW2* and *AaTAR2* were all highly expressed in young leaves and showed low expression in mature leaves, which is consistent with the expression pattern of *AaMYC3* (Figures 2c and S6h; Yan *et al*., 2017; Zhou *et al*., 2020). These RT‐qPCR results aligned with RNA‐seq data (Figure 4e,f), suggesting *AaHD1* as a potential AaMYC3 target. Subsequent dual‐luciferase assays in tobacco leaves, with AaMYC3 as the effector and the promoter of *AaHD1* driving a luciferase reporter (Figure 5c), demonstrated that AaMYC3 significantly upregulated the activity of *AaHD1* promoter, enhancing LUC:REN ratios by 1.85‐ to 2.41‐fold over the control (Figure 5d). This activation was corroborated by luciferase luminescence assays (Figure 5e).

To confirm whether AaMYC3 directly binds to the promoter of *AaHD1*, yeast one‐hybrid (Y1H) assays were performed (Figure 5f). The results indicate that AaMYC3 binds to the G‐box (CACGTG) *cis*‐element (Figure S10) within the *AaHD1* promoter. Notably, the binding signal diminishes upon mutation of the G‐box motif to the CTTTTTG sequence (Figure 5 g). To confirm the binding effect *in vitro*, we obtained the MBP‐AaMYC3 recombinant protein (Figure S11) to perform electrophoretic mobility shift assays (EMSAs). The results showed that AaMYC3 was able to directly bind the G‐box *cis*‐element of the *AaHD1* promoter *in vitro* (Figure 5 h). When the G‐box (CACGTG) *cis*‐elements were mutated to the CTTTTG sequences, the binding signal disappeared. To verify this interaction within *A. annua*, CUT&Tag‐qPCR assays were performed on OE‐*AaMYC3* plants bearing a Flag tag. Quantitative PCR of the enriched CUT&Tag nucleic acid library showed significant enrichment at the *AaHD1* promoter, confirming *AaHD1* as a direct downstream target of AaMYC3 *in vivo* (Figure S12). Taken together, these results provide evidence that AaMYC3 enhances *AaHD1* transcription by specifically targeting the G‐box *cis*‐element in the *AaHD1* promoter, thereby increasing GST density in *A. annua*.

### AaMYC3 directly regulates artemisinin biosynthesis by activating the *CYP71AV1* and *ALDH1* gene expression

**Figure 6:**
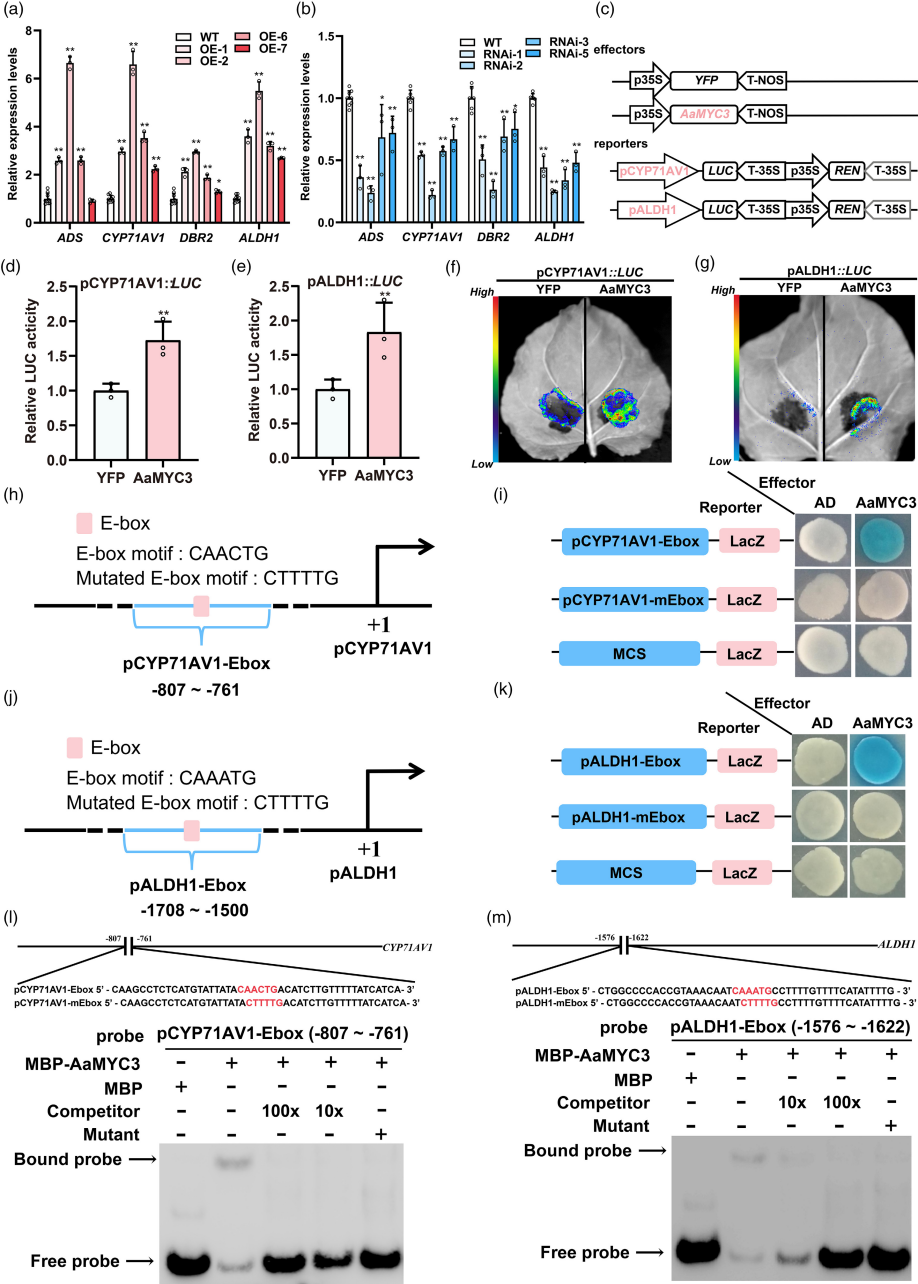
AaMYC3 positively regulates artemisinin biosynthesis by directly activating *CYP71AV1* and *ALDH1* transcription. (a, b) Relative expression levels of artemisinin biosynthetic genes in OE‐*AaMYC3* and RNAi‐*AaMYC3* lines compared with wild‐type (WT) *Artemisia. annua*, respectively. (c) Structural diagrams of effectors (35S::*YFP* and 35S::*AaMYC3*)and reporters (pCYP71AV1::*LUC* and pALDH1::*LUC*) in dual‐luciferase (Dual‐LUC) assays (REN, renilla luciferase; LUC, firefly luciferase). (d, e) The activities of *CYP17AV1* and *ALDH1* promoters fused to the LUC reporters were determined by Dual‐LUC assays. Relative LUC activities were normalized to the reference REN. Yellow fluorescent protein (YFP) effector was used as a negative control, and the LUC: REN ratios of YFP were set as 1. (f, g) LUC chemiluminescence imaging system photographed fluorescence in Dual‐LUC assays. (h, j) Schematic diagrams of the position of fragments on the promoters of *CYP71AV1* and *ALDH1* used in yeast one‐hybrid (Y1H) assays. The positions of potential response E‐box *cis*‐elements are shown as rounded square boxes, and they are numbered according to their distance from the translation initiation site (ATG), which is set as +1. (i) Y1H assays between AaMYC3 and pCYP71AV1‐Ebox or pCYP71AV1‐mEbox, of which the E‐box (CAACTG) motif was mutated to the mE‐box (CTTTTG) motifs. MCS: multiple cloning sites. The data represent five independent experiments, and representative results are shown. (k) Y1H assays between AaMYC3 and pALDH1‐Ebox or pALDH1‐mEbox, of which the E‐box (CAAATG) motif was mutated to the mE‐box (CTTTTG) motifs. MCS: multiple cloning sites. The data represent five independent experiments, and representative results are shown. (l, m) Electrophoretic mobility shift assays showed the binding of MBP‐AaMYC3 to the E‐box of *CYP71AV1* (l) and *ALDH1* (m) promoter, respectively. MBP protein was used as a negative control. Unlabeled pCYP71AV1‐Ebox and pALDH1‐Ebox at 1×, 10×, and 50× molar ratios as competitors. The E‐box is highlighted in red. All data represent the means ± SDs (*n* ≥ 3 as indicated in the figure; *P < 0.05; **P < 0.01; Student's *t*‐test).

**Figure 7:**
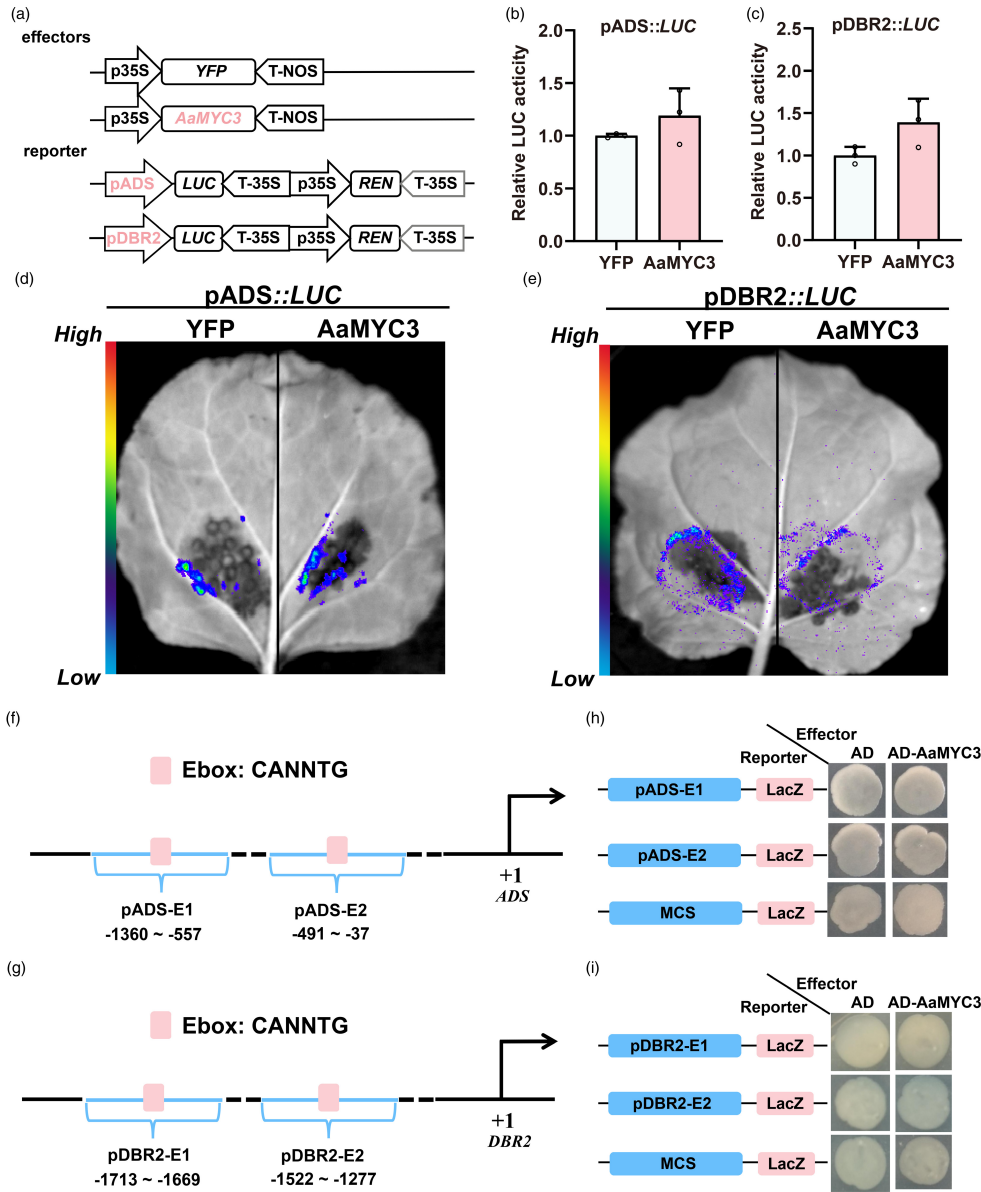
AaMYC3 can not bind to and activate the promoters of *ADS* and *DBR2*. (a) Structural diagrams of effectors (35S::*YFP* and 35S::*AaMYC3*) and reporters (pADS::*LUC* and pDBR2::*LUC*) in dual‐luciferase (Dual‐LUC) assays (REN, renilla luciferase; LUC, firefly luciferase). (b‐c) The activities of *ADS* and *DBR2* promoters fused to the LUC reporters were determined by Dual‐LUC assays. Relative LUC activity was normalized to the reference REN. Yellow fluorescent protein (YFP) effector was used as a negative control, and the LUC: REN ratios of YFP were set as 1. All data represent the means ± SDs (*n* ≥ 3 as indicated in the figure; Student's *t*‐test). (d‐e) LUC chemiluminescence imaging system photographed fluorescence in Dual‐LUC assays. (f, h) Schematic diagrams of the position of fragments on the promoters of *DBR2* and *ALDH1* used in yeast one‐hybrid (Y1H) assays. The positions of potential response E‐box *cis*‐elements are shown as rounded square boxes, and they are numbered according to their distance from the translation initiation site (ATG), which is set as +1. (g, i) Y1H assays between AaMYC3 and pADS‐Ebox or pDBR2‐Ebox. MCS: multiple cloning sites. The data represent five independent experiments, and representative results are shown.

Dihydroartemisinic acid (DHAA), a direct product catalysed by artemisinin biosynthesis enzymes, exhibited an up‐regulation of 1.23‐ to 1.65‐fold in OE‐*AaMYC3* lines compared to the wild type (Figure S13). Similarly, the expression levels of *ADS*, *CYP71AV1*, *DBR2*, and *ALDH1* were significantly elevated, ranging from 2.58‐ to 6.65‐fold, 2.23‐ to 6.59‐fold, 1.23‐ to 2.94‐fold, and 2.69‐ to 5.48‐fold, respectively (Figure 6a). Conversely, DHAA content in RNAi‐*AaMYC3* lines decreased by 29–49% relative to the control (Figure S13), leading to a substantial suppression of *ADS*, *CYP71AV1*, *DBR2*, and *ALDH1* expression levels by 28–77%, 33–78%, 25–74%, and 52–75%, respectively (Figure 6b). Considering the enriched sesquiterpenoid biosynthesis KEGG pathway (Figure 4b,d) and the expression profiles of genes from the artemisinin biosynthesis pathway in RNA‐seq data (Figure 4e,f), we propose that AaMYC3 likely regulates the transcription of these four genes. Dual‐LUC assays were performed in tobacco leaves. *AaMYC3* was expressed as an effector in the same manner, and the promoters of four artemisinin biosynthetic genes were individually inserted into the pGreen0800‐LUC plasmid to generate reporters. Each pair of effectors and reporters was transiently co‐expressed in tobacco (Figure 6c). The results of dual‐LUC assays showed that AaMYC3 significantly activated the promoters of *CYP71AV1* and *ALDH1*, resulting in LUC:LEN ratios of pCYP71AV1::LUC and pALDH1::LUC ranging from 1.45 to 2.00‐fold and 1.38 to 2.32‐fold, respectively (Figure 6d,e). The activation of AaMYC3 on pCYP71AV1 and pALDH1 was further confirmed by LUC luminescence assays (Figure 6f,g). However, AaMYC3 did not influence the transcription of *ADS* and *DBR2* (Figure 7a–e).

As a member of the bHLH TFs, AaMYC3 is hypothesised to bind the E‐box (CANNTG) motif within the promoters of four specific genes. To investigate this, yeast one‐hybrid (Y1H) assays were conducted. The data indicated that AaMYC3 directly interacts with the E‐box present in the promoters of *CYP71AV1* and *ALDH1* (Figures 6h–k and S14). The binding effect was abolished when these *cis*‐elements were mutated to the CTTTTG sequence (Figures 6h–k and S14). However, AaMYC3 could not bind to the *ADS* and *DBR2* promoters (Figure 7f–i). The further EMSAs confirmed that AaMYC3 recombinant protein binds pCYP71AV1‐Ebox and pALDH1‐Ebox fragments *in vitro* (Figure 6l,m). When the E‐box (CANNTG) were mutated to the CTTTTG sequences, the binding signals disappeared. Further in *vivo* analysis was performed using OE‐*AaMYC3*‐Flag lines to examine protein‐DNA interactions through CUT&Tag‐qPCR assays. The results demonstrated a significant enrichment of AaMYC3 within the E‐box‐containing promoter regions of *CYP71AV1* and *ALDH1* in *A. annua* (Figure S15). These findings support the notion that AaMYC3 modulates artemisinin biosynthesis by directly binding to and activating the transcription of *CYP71AV1* and *ALDH1*.

### AaMYC3 indirectly regulates artemisinin biosynthesis through interaction with AabHLH1 and AabHLH113

**Figure 8:**
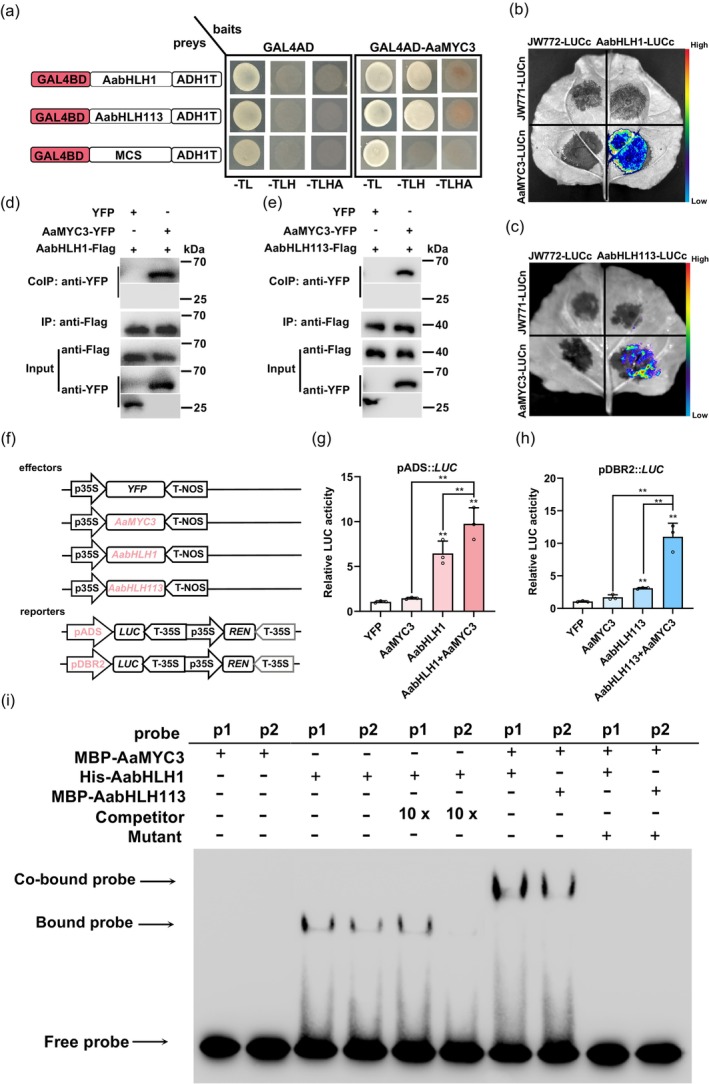
AabHLH1 and AabHLH113 interact with and enhance the transcriptional activities of AaMYC3 on *ADS* and *DBR2* promoters, respectively. (a) Y2H assays showing the interactions AaMYC3 with AabHLH1 or AabHLH113. Left schematic representations of the structure of AabHLH1 and AabHLH113 protein fusion binding domain (GAL4BD, highlighted red) as baits used in Y2H assays. AaMYC3 protein was fused onto the activation domain as prey. The data represent five independent experiments, and representative results are shown. (b, c) BiLC assays show the interaction between AaMYC3 with AabHLH1 or AabHLH113. LUC chemiluminescence imaging system photographed fluorescence and the intensities indicate the degree of binding capacity in BiLC assays. (d, e) Co‐IP assays show that AaMYC3 interacts with AabHLH1 or AabHLH113 *in vivo*. YFP was used as a negative control. Protein extracts before (Input) immunoprecipitation were detected by western blot with anti‐Flag and anti‐YFP antibodies, respectively. Protein extracts after immunoprecipitation with anti‐Flag antibody‐conjugated beads were detected by western blot with anti‐Flag antibody (IP) and anti‐YFP antibody (Co‐IP), respectively. (f) Structural diagrams of effectors (35S::*AabHLH1*, 35S::*AabHLH113* and 35S::*AaMYC3*) and reporters (pADS::*LUC* and pDBR2::*LUC*) in dual‐luciferase (Dual‐LUC) assays (REN, renilla luciferase; LUC, firefly luciferase). (g, h) The activities of *ADS* and *DBR2* promoters fused to the LUC reporters were determined by Dual‐LUC assays. Relative LUC activity was normalized to the reference REN. Yellow fluorescent protein (YFP) effector was used as a negative control, and the LUC: REN ratios of YFP were set as 1. All data represent the means ±SDs (*n* ≥ 3 as indicated in the figure; **P < 0.01; Student's *t*‐test). (i) Electrophoretic mobility shift assays (EMSAs) showed the binding of His‐AabHLH1 and His‐AabHLH113 to the E‐box and mE‐box of *ADS* and *DBR2* promoter, respectively. EMSA also showed these binding, after MBP‐AaMYC3 interacted with His‐AabHLH1 and His‐AabHLH113, respectively. The p1 and p2 were labelled pADS‐3 × Ebox/pADS‐3 × mEbox and pDBR2‐Ebox/pDBR2‐mEbox, as the method described, respectively. Unlabeled pADS‐3 × Ebox and pDBR2‐Ebox at 10× molar ratios as competitors. All probe sequences are placed in Table S7. [Correction added on 13 September 2024, after first online publication: the Figures 5a and 8i are revised in this version.]

AaMYC3 was unable to activate the promoters of *ADS* and *DBR2* in tobacco (Figure 7); however, the transcript levels of *ADS* and *DBR2* varied in tandem with the expression of *AaMYC3* in both OE‐*AaMYC3* and RNAi‐*AaMYC3* lines (Figures 4e,f and 6a,b). Consequently, it is hypothesised that AaMYC3 may indirectly influence the transcription of *ADS* and *DBR2*, possibly through the involvement of other factors. To investigate this possibility, IP‐MS assays were performed on OE‐*AaMYC3* transgenic lines to identify proteins that interact with AaMYC3. A total of 21 candidate proteins were identified, with approximately half belonging to the bHLH gene family (Table S2). Given that AabHLH1 and AabHLH113 have been recognized as positive regulators of artemisinin biosynthesis (Li *et al*., 2019; Yuan *et al*., 2023), they were selected to determine whether AaMYC3 functions in conjunction with other bHLH proteins (Figure S16). To confirm this speculation, the yeast two‐hybrid (Y2H) assays were performed. The results revealed that AaMYC3 physically interacts with AabHLH1 and AabHLH113 (Figure 8a).

To further confirm the interactions between AaMYC3 and AabHLH1 or AabHLH113, bimolecular luciferase complementation assays (BiLC) were performed. When the fusion proteins of AaMYC3‐LUCn and AabHLH1‐LUCc and AabHLH113‐LUCc, respectively, were co‐expressed in tobacco, strong relative LUC activity was detected, whereas no LUC signal was observed when the AabHLH1‐LUCc and AabHLH113‐LUCc were expressed alone (Figure 8b,c). Co‐immunoprecipitation (Co‐IP) assays further validated the interactions between AaMYC3 and AabHLH1, as well as AabHLH113 (Figure 8d,e).

Previous studies have shown that bHLH transcription factors tend to form homo‐ or heterodimers that function together (Schweizer *et al*., 2013; Swinnen *et al*., 2022). Thus, Dual‐LUC assays were performed using the effectors (35S::*YFP*, 35S::*AaMYC3*, 35S::*AabHLH1* and 35S::*AabHLH113*) along with the reporters (pADS::*LUC* and pDBR2::*LUC*) (Figure 8f). Compared to YFP controls, AabHLH1 and AabHLH113 significantly increased the activities of *ADS* and *DBR2* promoters (Figure 7g,h), which is consistent with previous reports (Ji *et al*., 2014; Yuan *et al*., 2023). Importantly, the LUC:REN values of pADS::*LUC* and pDBR2::*LUC* increased 1.33‐ to 1.80‐fold and 2.73‐ to 4.28‐fold when AaMYC3 was co‐expressed simultaneously with AabHLH1 and AabHLH113, compared to AabHLH1 and AabHLH113 expressed individually, respectively (Figure 8g,h). Consistent with the previous results made by Ji *et al*. (2014) and Yuan *et al*. (2023), AabHLH1 and AabHLH113 bind to the promoter of *ADS* and *DBR2*, respectively (Figure 8i). More importantly, EMSAs also showed that the presence of AaMYC3 enhanced the binding ability of AabHLH1 and AabHLH113 to their targets, respectively (Figure 8i). In summary, these findings indicate that AaMYC3 interacts with AabHLH1 and AabHLH113 to promote the transcription of the *ADS* and *DBR2*, thereby regulating artemisinin biosynthesis.

## Discussion

JA, as a crucial phytohormone, plays significant roles in regulating plant development and secondary metabolism. Regarding plant trichomes, JA induces the initiation of trichome formation in *Arabidopsis* and *A. annua* (Ma *et al*., 2021; Song *et al*., 2022a), as well as the elongation of trichomes in cotton and tomato (Hu *et al*., 2016; Hua *et al*., 2021). Modulating the expression of the JA biosynthetic pathway gene *AOS* may enhance the quantity of trichomes (Wu *et al*., 2008). JAZ proteins, as negative regulators of the jasmonate pathway, can suppress the development of trichomes by inhibiting MYB‐like proteins in *Arabidopsis* and cotton (Hu *et al*., 2016; Song *et al*., 2022a). In terms of secondary metabolism, JA modulates the biosynthesis of phenolic secondary metabolites such as anthocyanins, flavonols, lignins, and volatile phenolic compounds through MYB transcription factors (De Geyter *et al*., 2012). For instance, JA induces biosynthesis of anthocyanin and proanthocyanidin by mediating the JAZ1‐TRB1‐MYB9 complex in apple (An *et al*., 2021). AP2/ERFs play significant roles in JA‐regulated alkaloid and terpenoid synthesis, as exemplified by the jasmonate‐responsive AP2/ERF transcription factors AaERF1 and AaERF2 positively regulating artemisinin biosynthesis in *A. annua* (Yu *et al*., 2012). GSTs serve as biosynthetic factories for plant secondary metabolism (Xiao *et al*., 2016). The biosynthesis of plant secondary metabolites is intricately linked to their growth and development (McConkey *et al*., 2000). For instance, the MYC2‐like transcription factor GoPGF in cotton, which simultaneously regulates gland size and secondary metabolite synthesis, holds significant implications for molecular breeding aimed at enhancing cotton resistance and seed utilization (Wang *et al*., 2024). It is also found that GoPGS acts as a negative regulator of pigment gland development downstream of GoPGF through the JA signalling pathway. Silencing GoPGS increased the size of pigmented glands, thereby enhancing the biosynthesis and accumulation of gossypol‐related terpenoids (Sun *et al*., 2024). Due to the dual roles of JA in regulating gland size and secondary metabolism, as evidenced by GhVQ22 and JAVL repressing GoPGF transcription and thereby affecting gland initiation and secondary metabolic accumulation in cotton (Wang *et al*., 2024; Wu *et al*., 2024b; Zhang *et al*., 2024), there is immense potential for pathway genes to concurrently regulate metabolism and glandular trichome development, particularly the MYC2‐like transcription factors as core genes in this pathway. This conjecture is also supported in tomato, where JA‐induced SlMYC1 regulates not only terpenoid biosynthesis but also the formation of Type VI glandular trichomes (Xu *et al*., 2018). After JA treatment in *A. annua*, GST density significantly increased after 7 days. Artemisinin production also significantly increased 12 h post‐treatment, suggesting that the JA pathway can simultaneously regulate GST development and artemisinin metabolism (Figure S17). Through IP‐MS screening (Figure S1), we identified *AaMYC3* induced by JA, and transgenic results show phenotypes with concurrent changes in GST and artemisinin (Figures 1, 2, 3), providing new target genes for the coordinated regulation of trichome development in linked secondary metabolism.

Focusing on *A. annua*, the regulatory mechanisms of artemisinin biosynthesis induced by JA have been elucidated (Zheng *et al*., 2023). AabHLH1, a JA‐responsive transcription factor, positively regulates artemisinin biosynthesis by directly binding to and activating the *ADS* promoter (Ji *et al*., 2014). Similarly, AabHLH113 enhances artemisinin accumulation by activating *DBR2* and *ALDH1* transcription (Yuan *et al*., 2023). The density of GSTs is also induced by JA (Figure S17), which stimulates artemisinin production in *A. annua* (Xie *et al*., 2021b). JA‐sensitive AaHD1 positively controls GST initiation, and AaHD1 interacts with AaJAZ8, suggesting that JA‐mediated GST initiation is mediated by controlling the transcriptional activity of AaHD1 (Yan *et al*., 2017). The expression of both *AaHD1* and its downstream target genes, *AaGSW2* and *AaTAR2*, was also induced by JA to varying degrees (Figure S17). AaMYB16 interacts with AaHD1 to promote GST initiation, but JA‐induced AaMYB5 competes with AaMYB16 to interact with AaHD1 and inhibit GST formation (Xie *et al*., 2021b). This suggests that there is a feedback regulatory mechanism for JA‐mediated GST development to maintain GST density within a certain range in *A. annua*. However, no transcription factor has been identified to concurrently regulate the expression of all four enzymes in the artemisinin‐specific pathway while also controlling the initiation of GSTs, leading to the regulatory mechanisms induced by JA between the two remaining disjointed.

**Figure 9:**
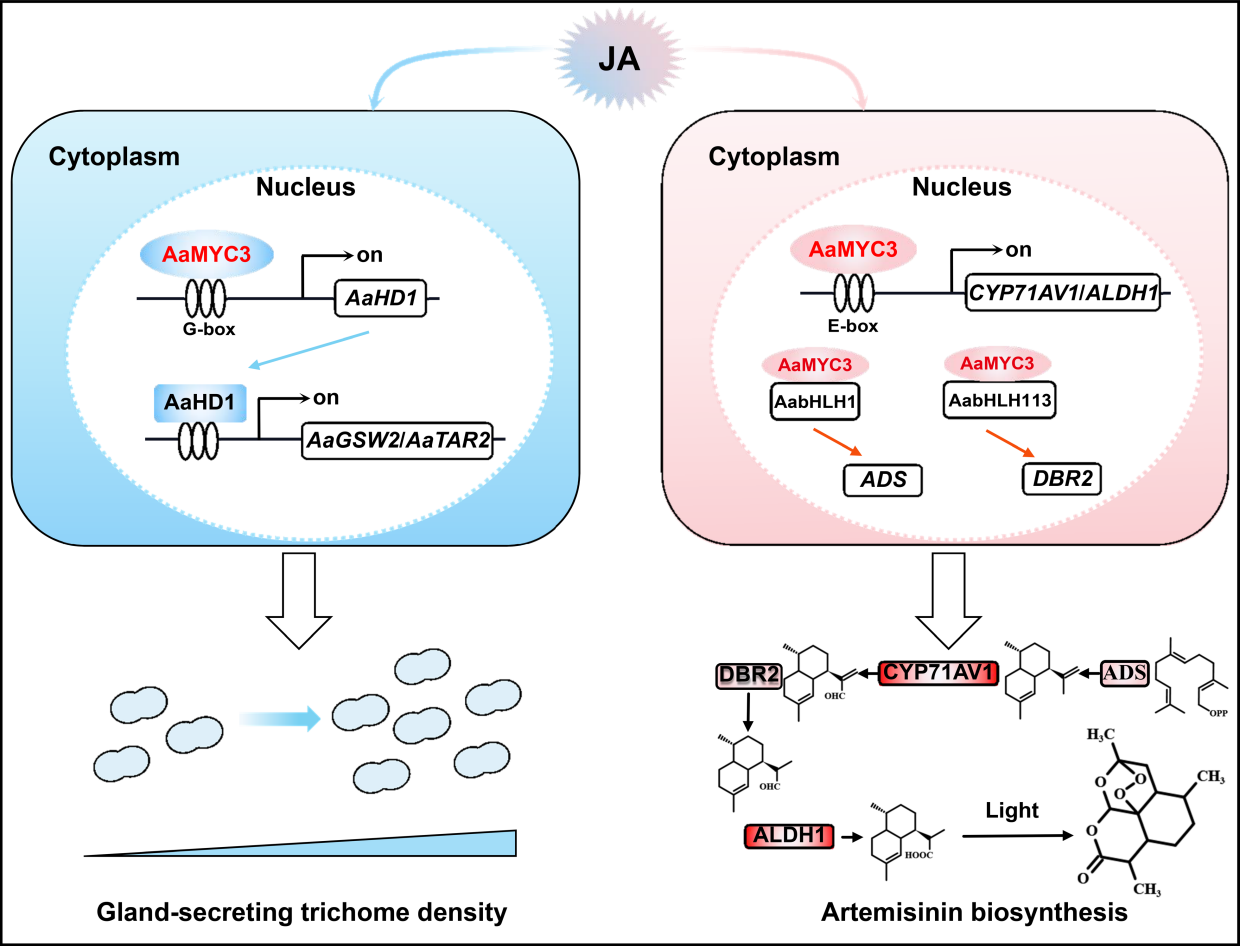
The model that AaMYC3 regulates both gland‐secreting trichome (GST) density and artemisinin biosynthesis in *Artemisia annua*. AaHD1 is a positive regulator of *A. annua* GST development and AaMYC3 regulates GST density by directly binding to and activating the G‐box *cis*‐element of the *AaHD1* promoter in *A. annua*. Meanwhile, AaMYC3 directly regulates artemisinin biosynthetic genes *CYP71AV1* and *ALDH1* transcription, thereby regulating artemisinin biosynthesis. AabHLH1 and AabHLH113 promote artemisinin accumulation through direct activation of the promoters of *ADS* and *DBR2*, respectively. AaMYC3 interacts with AabHLH1 and AabHLH113, respectively, and enhances the transcriptional activation of *ADS* and *DBR2*. The blue colour represents the modulation of GST density. Pink represents the regulation of artemisinin biosynthesis. Blue and pink thin arrows represent post‐transcriptional translation into proteins. Wide arrows represent the process of change. Blue and pink curved arrows indicate JA induction. Black broken arrows indicate gene transcription. Double ellipsoids represent GST. Triangles represent GST density trends. In the artemisinin biosynthesis pathway, red boxes indicate enzymes directly regulated by AaMYC3, pink boxes indicate enzymes indirectly regulated by AaMYC3, and short black arrows indicate enzymatic reaction steps. AaMYC3 is highlighted in red.

Data from molecular and genetic assays indicate that JA‐induced AaMYC3 (Figure 2) acts as a positive regulator to modulate GST density (Figure 3) by directly binding to and activating the G‐box on the *AaHD1* promoter (Figures 4, 5 and S12) *in vitro* and *in vivo*. AaHD1, a JA‐mediated GST initiation key factor, directly regulates *AaGSW2* and *AaTAR2* transcription (Xie *et al*., 2021a; Zhou *et al*., 2020). It was found that JA‐induced AaMYC3 directly regulates the transcription of *AaHD1* (Figures 3 and 5), and RNA‐seq showed that compared with control in OE‐*AaMYC3* and RNAi‐*AaMYC3* transgenic *A. annua*, both *AaGSW2* and *AaTAR2* transcripts were both significantly higher and lower, respectively (Figure 4). This suggests that AaMYC3 may regulate the expression of *AaGSW2* and *AaTAR2* through AaHD1 to promote the increase of GST density by this cascade regulatory mechanism, which would be checked by genetic assays in the future work. It is noted that JA‐induced AaSPL1 and AaHD8 directly activate the “GTAC” and L‐box motifs on the *AaHD1* promoter, respectively (He *et al*., 2022; Yan *et al*., 2018). The difference in the binding sites between AaSPL1, AaHD8, and AaMYC3 suggests that AaMYC3 regulates *AaHD1* transcription through a distinct signalling pathway (Figure 5f–h). In addition, AaMYC3 directly activates the expression of artemisinin biosynthetic genes, *CYP71AV1* and *ALDH1* (Figure 6), suggesting that AaMYC3 is an essential regulatory node for both GST density and artemisinin biosynthesis (Figure 9).

JA‐induced AabHLH1 activates *ADS* and *CYP71AV1* promoters, increasing artemisinin biosynthesis (He *et al*., 2022; Yan *et al*., 2018). Conversely, JA‐responsive AabHLH113 directly activates *DBR2* and *ALDH1* transcription, enhancing artemisinin accumulation, but could not bind to *ADS* and *CYP71AV1* promoters (Xie *et al*., 2021b). Interestingly, the expression of *ADS* and *DBR2* coincides with *AaMYC3* in transgenic *A. annua*, suggesting the involvement of other TFs in their regulation (Figure 7). Our results showed that AaMYC3 interacts with AabHLH1 and AabHLH113 *in vitro* and *in vivo* (Figures 8a–e and S16), which enhances the transcriptional activation of *ADS* by AabHLH1 and *DBR2* expression by AabHLH113 (Figure 8f–h). Not only that, EMSA results showed that AaMYC3 also enhanced the binding ability of AabHLH1 and AabHLH113 to the *ADS* and *DBR2* promoters, respectively (Figure 8i). This suggested that the regulation of *ADS* and *DBR2* transcription by AaMYC3 is dependent on AabHLH1 and AabHLH113, and AaMYC3 is a key factor that boosts the expression of all four genes located on the artemisinin‐specific biosynthesis route simultaneously (Figure 9), which act as a TF to transcribe *ADS* and *DBR2*, while serving as a co‐activator to enhance the function of AabHLH1 and AabHLH113 (Figure 9). These findings indicate that AaMYC3 serves as a crucial transcription factor located within the JA pathway capable of coordinating both GST development and artemisinin synthesis.

However, artemisinin synthesis in *A. annua* is not without constraints. Although *AaMYC3* overexpression does promote artemisinin biosynthesis and increase GST density, leading to a somewhat higher proportion of artemisinin in the leaves compared to the overexpression of genes like OE‐*AaMYC2* that solely regulate its synthesis (Shen *et al*., 2016), the effect on yield may not be merely cumulative. Specifically, the transcriptional up‐regulation of artemisinin biosynthetic genes in OE‐*AaMYC3* is proportionally less than that resulting from the overexpression of other reported positive regulators dedicated to artemisinin biosynthesis exclusively, such as *AaMYC2*，*AabZIP1*, *AaTCP15*, *AabHLH113*, etc. (Ma *et al*., 2021; Shen *et al*., 2016; Yuan *et al*., 2023; Zhang *et al*., 2015). Therefore, the increase in total artemisinin yield induced by the strategy that simultaneously controls GST density and metabolic intensity is not as substantial as expected when compared to strategies that overexpress artemisinin‐specific positive regulators. This suggests that plants may regulate *in vivo* levels of secondary metabolites to a certain extent, and an overabundance of artemisinin could likewise limit its further accumulation in *A. annua*. To date, no suitable technique has been identified that can demonstrate the proportionate increase in GST density alongside enhanced artemisinin biosynthesis, resulting in an up‐regulation of artemisinin. Our research primarily uncovers the molecular link between artemisinin biosynthesis and GST development, both regulated by the same gene‐*AaMYC3*, suggesting a genetic interconnection between plant secondary metabolism and growth. Emerging technologies such as CRISPR (Song *et al*., 2022b) and the use of nanoparticles in precision breeding (Zhao *et al*., 2022) may assist in fine‐tune the balance between GST density and artemisinin biosynthesis, thereby achieving the maximum yield. However, due to limitations imposed by transgenic technology, transformation receptors, and the lack of an efficient gene editing system for *A. annua*, significant constraints exist in constructing the regulatory network surrounding JA‐related gene like *AaMYC3* (Figure 9) or other genes regulating GST/artemisinin, thereby hindering the application of *A. annua* molecular breeding based on this. Despite the successful precedents of co‐expressing multiple genes in *A. annua* to study GST development and artemisinin synthesis, which boosts confidence in *A. annua* breeding and metabolic engineering, it remains time‐consuming and costly (Ma *et al*., 2018; Shi *et al*., 2017). Consequently, in future endeavours, establishing and optimizing *A. annua* gene editing systems, such as Cut‐Dip‐Budding applied in *A. annua* (Cao *et al*., 2024), and utilizing nanomaterials‐related delivery systems (Cao *et al*., 2023; Chen *et al*., 2022; Li *et al*., 2022), will facilitate the identification of regulatory modules modulating artemisinin biosynthesis and GST development, enabling the construction of molecular networks. This will, in turn, facilitate precise breeding practices aimed at increasing artemisinin yield.

## Author contributions

J.C. and M.Y. conceived and designed the entire research program. M.Y. completed most of the experiments. M.Y. and Y.S. culture transgenic materials. J.B. assisted in the completion of the Co‐IP assays. W.W. and B.N. watering plant material. J.C., J.W., and M.Y. wrote this article. All authors read and approved the final article.

## Conflicts of interest

The authors declare no conflict of interest.

## Supporting information


**Figure S1** The sequenced peptides were sequence‐aligned with the *A. annua* proteome and screened for AabHLH113 (d) and AaMYC3 (e).
**Figure S2** The entire heatmap of co‐expression analysis of bHLH transcription factors (TFs) in *Artemisia annua*.
**Figure S3** Phylogenetic analysis of 8 AabHLHs candidates together with all AtbHLH TFs from *Arabidopsis*.
**Figure S4** GUS histochemical staining assay showed *AaMYC3* expression position.
**Figure S5** Establishment of overexpressing *AaMYC3* transgenic *Artemisia annua* plants.
**Figure S6** Establishment of RNAi‐*AaMYC3* transgenic *Artemisia annua* plants.
**Figure S7** Transgenic *Artemisia annua* leaf phenotype and leaf sequence expression pattern of *AaGSW2*.
**Figure S8** Gene expression abundance statistics of OE‐*AaMYC3*, RNAi‐*AaMYC3*, and wild‐type *Artemisia annua* leaves.
**Figure S9** Differential gene analysis and Gene Set Enrichment Analysis (GSEA) of OE‐*AaMYC3* (OE), RNAi‐*AaMYC3* (RNAi), and wild‐type (WT) *Artemisia annua* leaves genes.
**Figure S10** Nucleotide sequences of *AaHD1* promoters.
**Figure S11** Acquisition of the recombinant protein MBP‐AaMYC3.
**Figure S12** CUT&tag‐qPCR identification of *AaHD1* directly regulated by AaMYC3 in OE‐*AaMYC3* transgenic *Artemisia annua*.
**Figure S13** Contents of dihydroartemisinic acid and HPLC chromatograms in transgenic *Artemisia annua* plants.
**Figure S14** Nucleotide sequences of *CYP71AV1* and *ALDH1* promoters.
**Figure S15** CUT&tag‐qPCR identification of *CYP71AV1* and *ALDH1* directly regulated by AaMYC3 in OE‐*AaMYC3* transgenic *Artemisia annua*.
**Figure S16** IP‐MS screening of proteins interacting with AaMYC3 in OE‐*AaMYC3* transgenic *Artemisia annua*.
**Figure S17** JA induced the content of GST density and artemisinin as well as the expression levels of *AaHD1*, *AaGSW2*, *AaTAR2* and artemisinin biosynthetic genes in *Artemisia annua*.
**Table S1** IP‐MS screening of 24 proteins interacting with AabHLH113.
**Table S2** IP‐MS screening of 21 proteins interacting with AaMYC3.
**Table S3** Sequences of primers used in molecular assays.
**Table S4** Sequences of primers used in yeast one‐hybrid assays.
**Table S5** Sequences of primers used in yeast two‐hybrid assays.
**Table S6** Sequences of primers used in co‐immunoprecipitation assays.
**Table S7** Sequences of probes used in electrophoretic mobility shift assays.

## Data Availability

The authors declare that all data supporting the findings of this study are available within the article and its Supplemental Information files or are available from the corresponding author upon request. The raw sequencing data of RNA‐seq were deposited in the NCBI (accession number: PRJNA1134801) and AaMYC3 (PQ007454).
