# Supplementary material for: AaMYC3 bridges the regulation of glandular trichome density and artemisinin biosynthesis in Artemisia annua
Source: Plant Biotechnol J. 2024 Aug 27;23(2):315–32. doi: 10.1111/pbi.14449 (PMC11772365; doi:10.1111/pbi.14449)
Supplement: Supplementary file 1 — Figure S1 The sequenced peptides were sequence‐aligned with the A. annua proteome and screened for AabHLH113 (d) and AaMYC3 (e). Figure S2 The entire heatmap of co‐expression analysis of bHLH transcription factors (TFs) in Artemisia annua. Figure S3 Phylogenetic analysis of 8 AabHLHs candidates together with all AtbHLH TFs from Arabidopsis. Figure S4 GUS histochemical staining assay showed AaMYC3 expression position. Figure S5 Establishment of overexpressing AaMYC3 transgenic Artemisia annua plants. Figure S6 Establishment of RNAi‐AaMYC3 transgenic Artemisia annua plants. Figure S7 Transgenic Artemisia annua leaf phenotype and leaf sequence expression pattern of AaGSW2. Figure S8 Gene expression abundance statistics of OE‐AaMYC3, RNAi‐AaMYC3, and wild‐type Artemisia annua leaves. Figure S9 Differential gene analysis and Gene Set Enrichment Analysis (GSEA) of OE‐AaMYC3 (OE), RNAi‐AaMYC3 (RNAi), and wild‐type (WT) Artemisia annua leaves genes. Figure S10 Nucleotide sequences of AaHD1 promoters. Figure S11 Acquisition of the recombinant protein MBP‐AaMYC3. Figure S12 CUT&tag‐qPCR identification of AaHD1 directly regulated by AaMYC3 in OE‐AaMYC3 transgenic Artemisia annua. Figure S13 Contents of dihydroartemisinic acid and HPLC chromatograms in transgenic Artemisia annua plants. Figure S14 Nucleotide sequences of CYP71AV1 and ALDH1 promoters. Figure S15 CUT&tag‐qPCR identification of CYP71AV1 and ALDH1 directly regulated by AaMYC3 in OE‐AaMYC3 transgenic Artemisia annua. Figure S16 IP‐MS screening of proteins interacting with AaMYC3 in OE‐AaMYC3 transgenic Artemisia annua. Figure S17 JA induced the content of GST density and artemisinin as well as the expression levels of AaHD1, AaGSW2, AaTAR2 and artemisinin biosynthetic genes in Artemisia annua. Table S1 IP‐MS screening of 24 proteins interacting with AabHLH113. Table S2 IP‐MS screening of 21 proteins interacting with AaMYC3. Table S3 Sequences of primers used in molecular assays. Table S4 Sequences of primers used in [file PBI-23-315-s001.docx]

**Supporting Information**

**
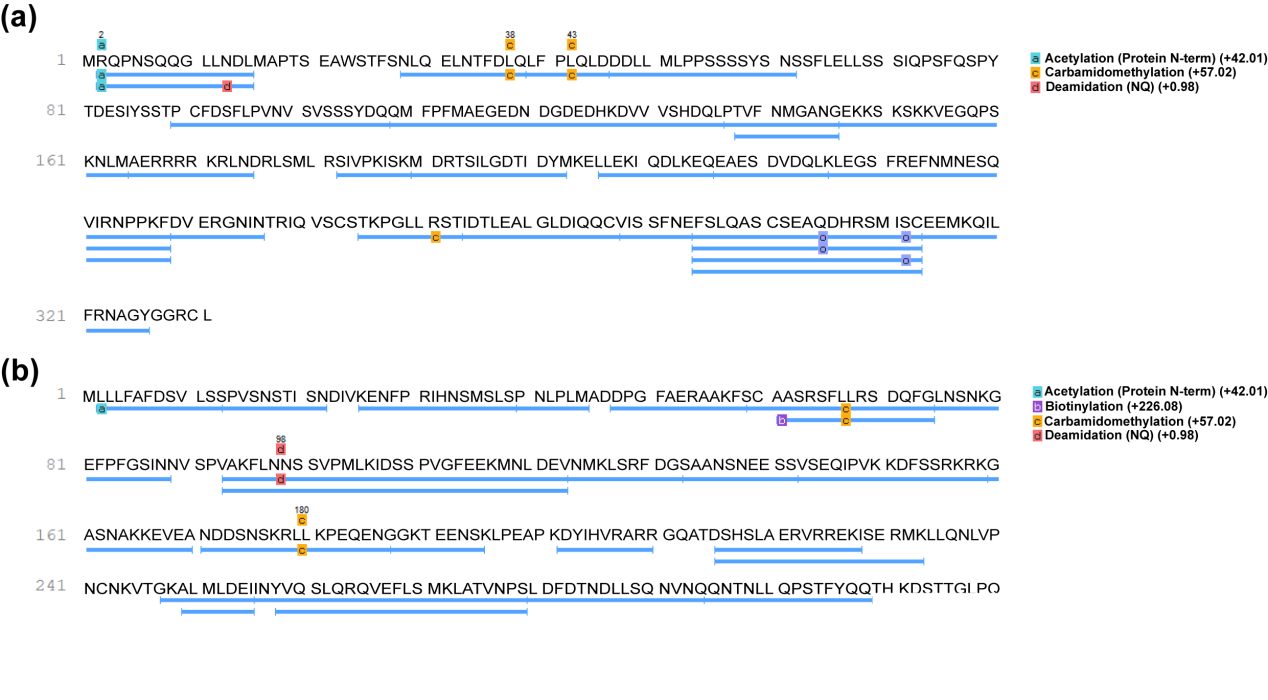
**

**Figure S1** The sequenced peptides were sequence-aligned with the *A. annua* proteome and screened for AabHLH113 (a) and AaMYC3 (b).

**
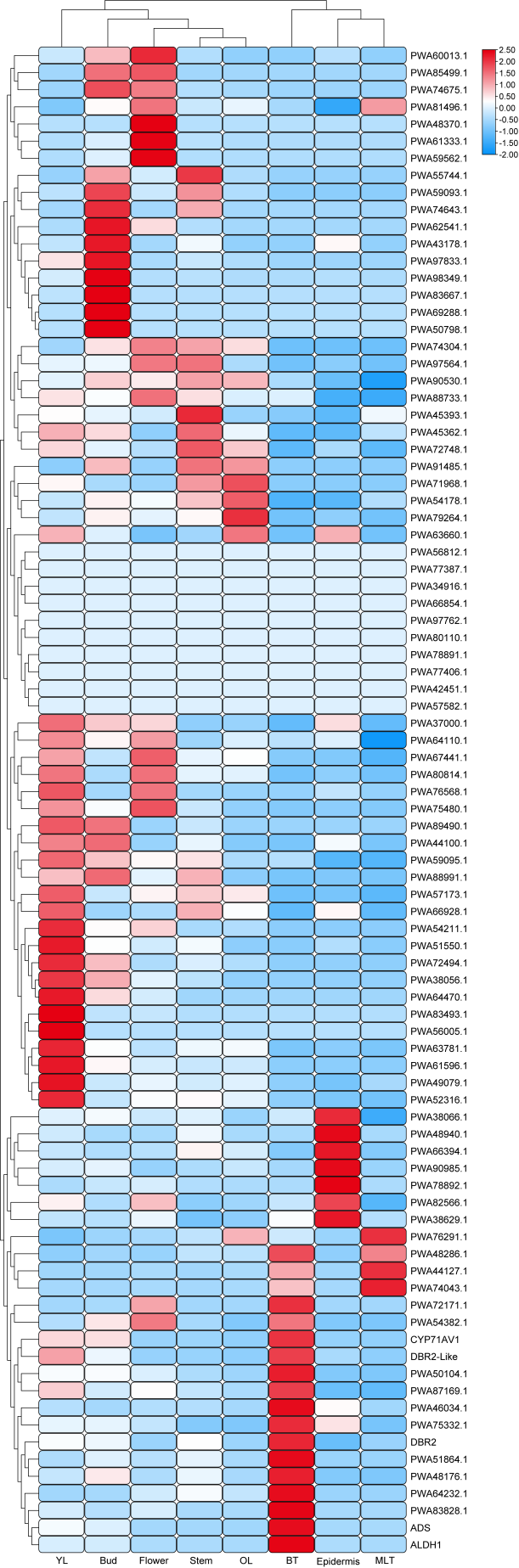
**

**Figure S2** The entire heatmap of co-expression analysis of bHLH transcription factors (TFs) in *Artemisia annua*. Young leaves (YL), buds, flowers, stem, old leaf (OL), bud trichomes (BT), epidermis and mature leaf trichomes (MLT).


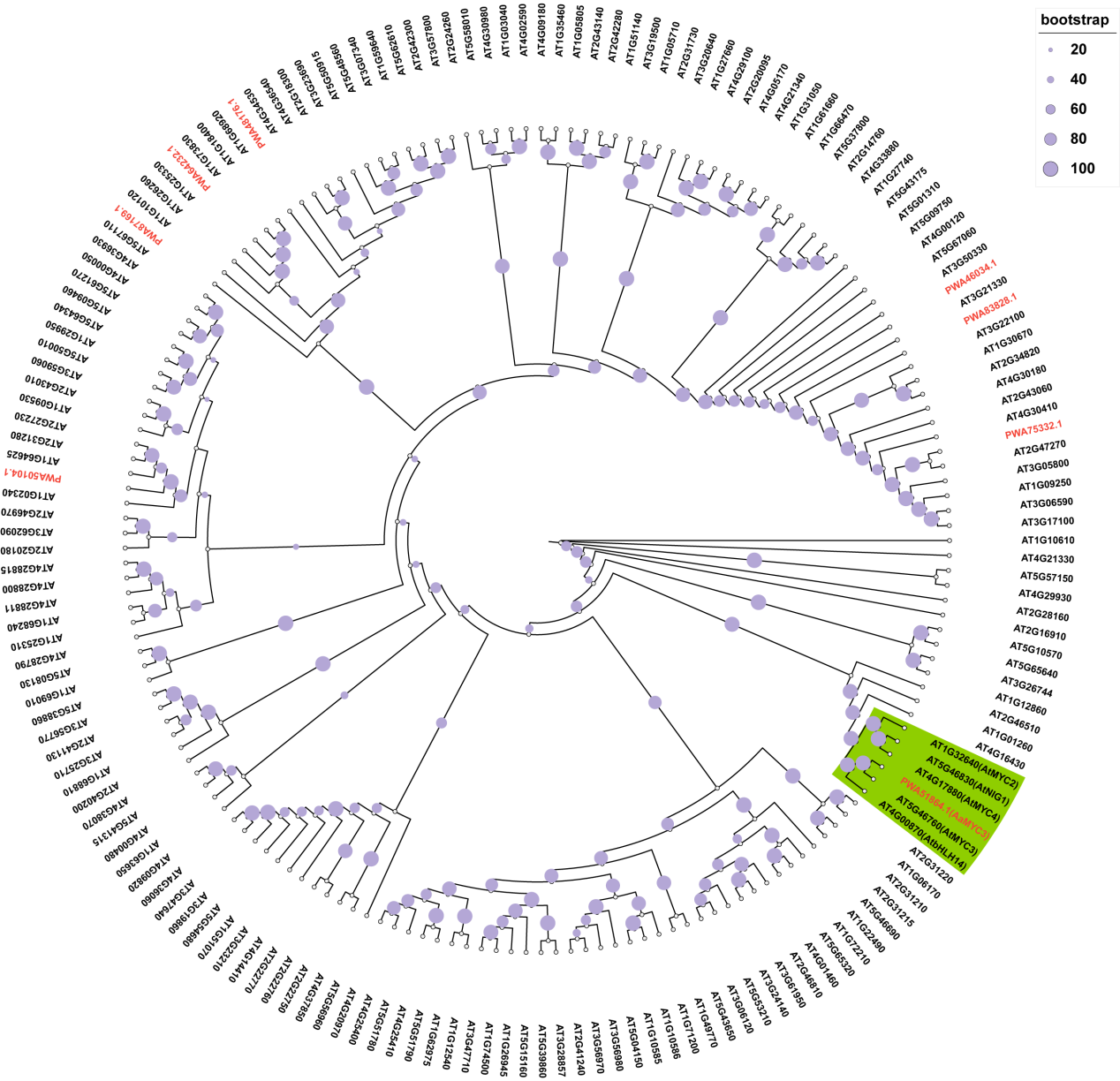


**Figure S3** Phylogenetic analysis of 8 AabHLHs candidates together with all AtbHLH TFs from *Arabidopsis*. Bootstrap values are shown as a light purple circle at branches of the maximum-likelihood tree. The 8 AabHLHs from *A. annua* in (Figure 1b) are highlighted with red; AaMYC3 and five AtbHLHs from *Arabidopsis* are highlighted with green.

**
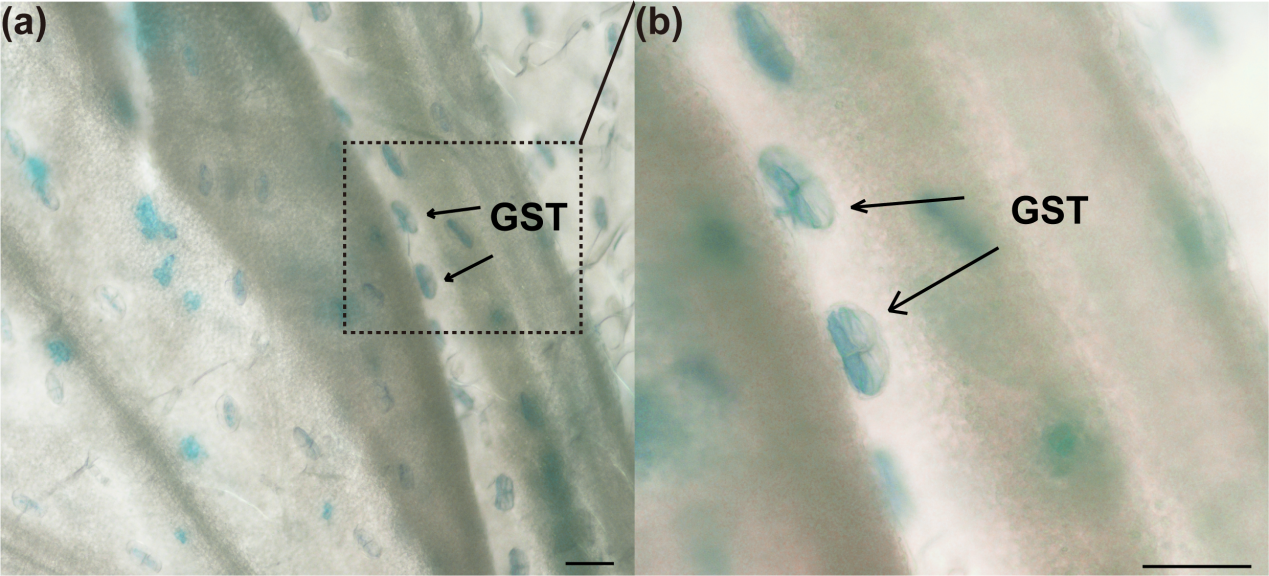
**

**Figure S4** GUS histochemical staining assay showed *AaMYC3* expression position. (a) (a) Microscopic image under 20× objective lens. (b) Microscopic image under 40× objective lens. gland-secreting trichomes (GSTs), Bar: 50 μm.

**
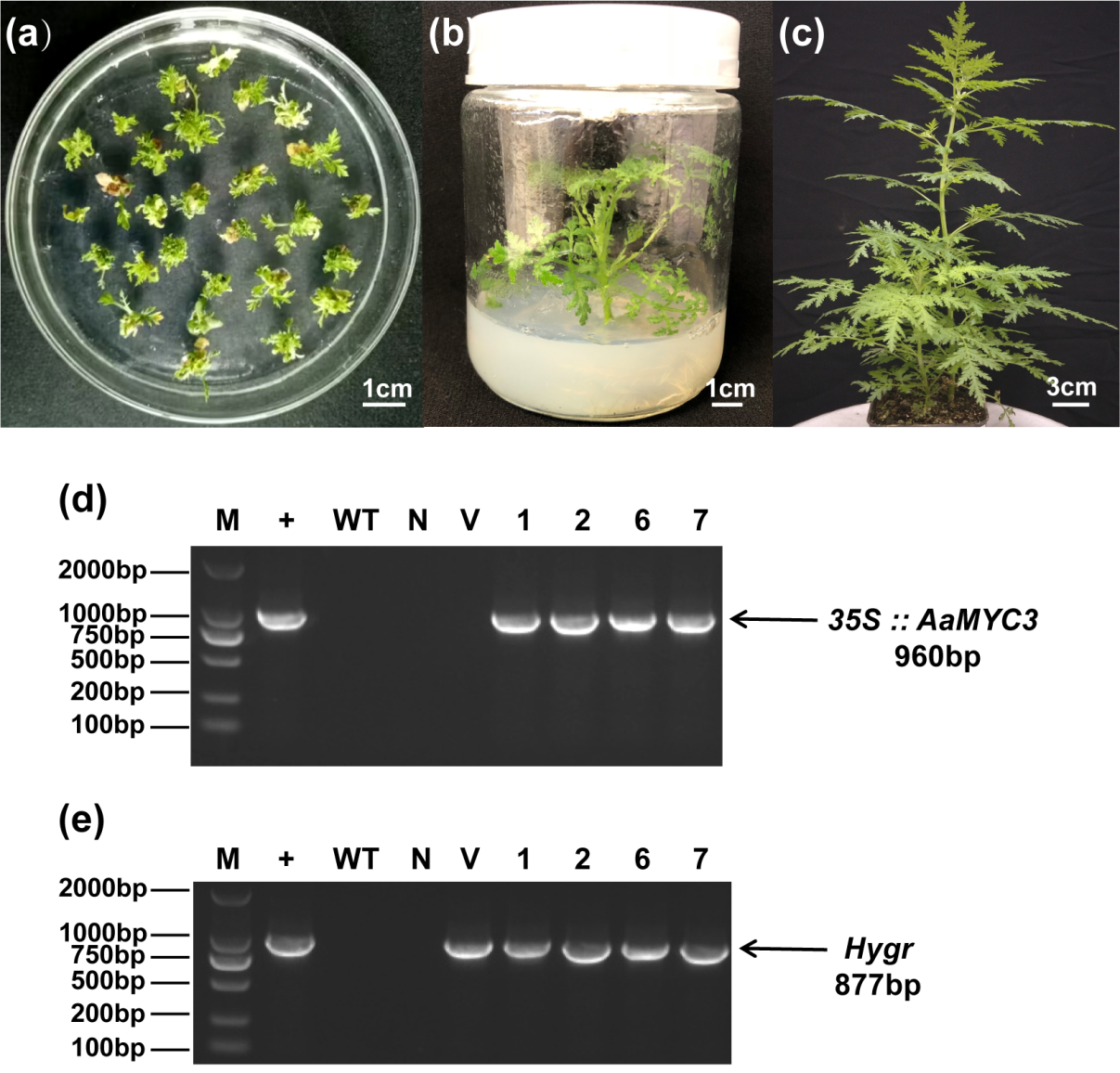
**

**Figure S5** Establishment of overexpressing *AaMYC3* transgenic *Artemisia annua* plants. (a-c) Germination, rooting and transplantation of transgenic *A. annua* plants overexpressing *AaMYC3*. (d) PCR identification of genomic DNA of transgenic *A. annua* plants. M: marker, +: positive control, WT: wild-type, N: negative control, V: empty vector.

**
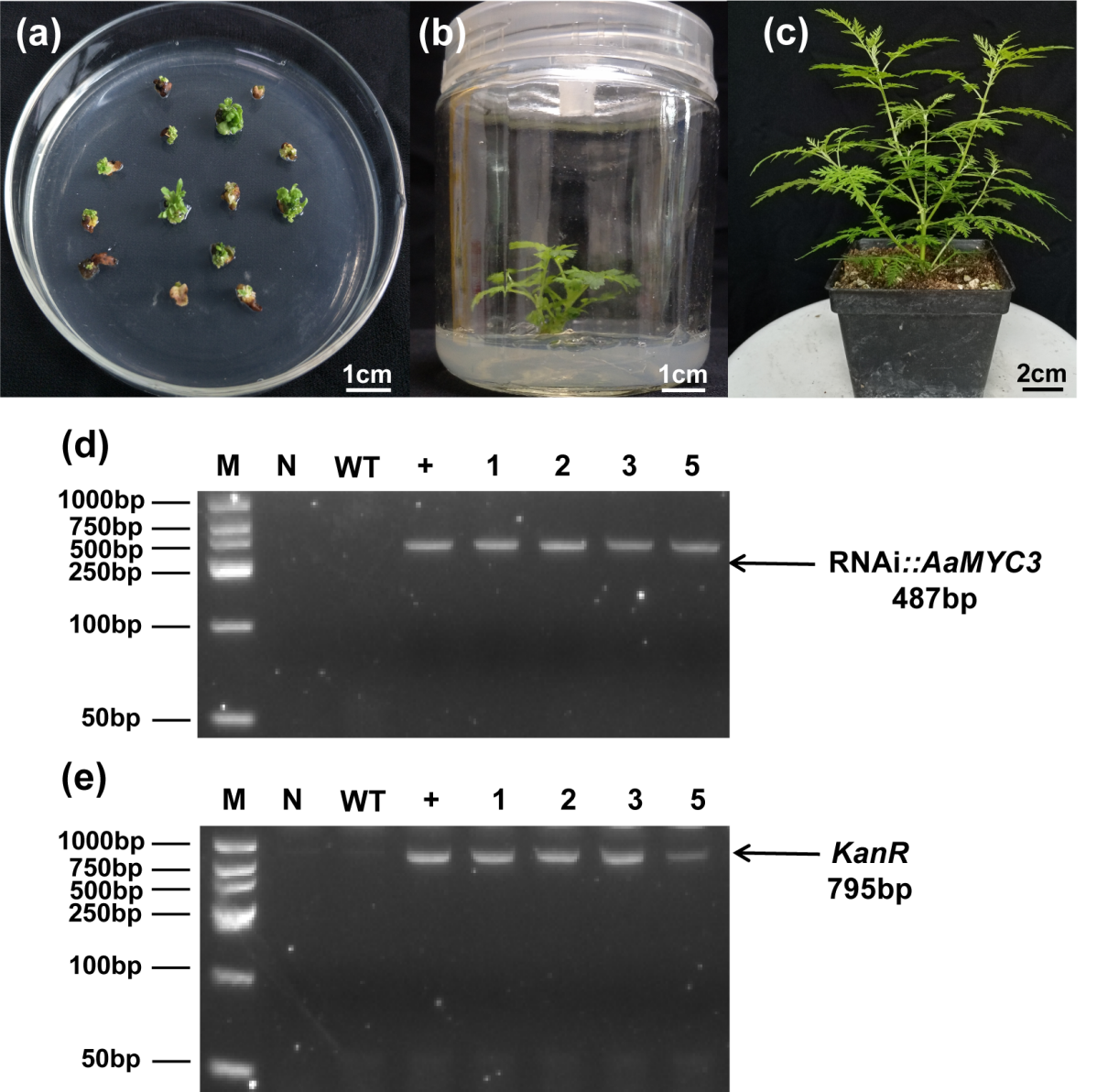
**

**Figure S6** Establishment of RNAi-*AaMYC3* transgenic *Artemisia annua* plants. (a-c) Germination, rooting and transplantation of RNAi-*AaMYC3* transgenic *A. annua* plants. (d) PCR identification of genomic DNA of transgenic *A. annua* plants. M: marker, +: positive control, WT: wild-type, N: negative control.

**
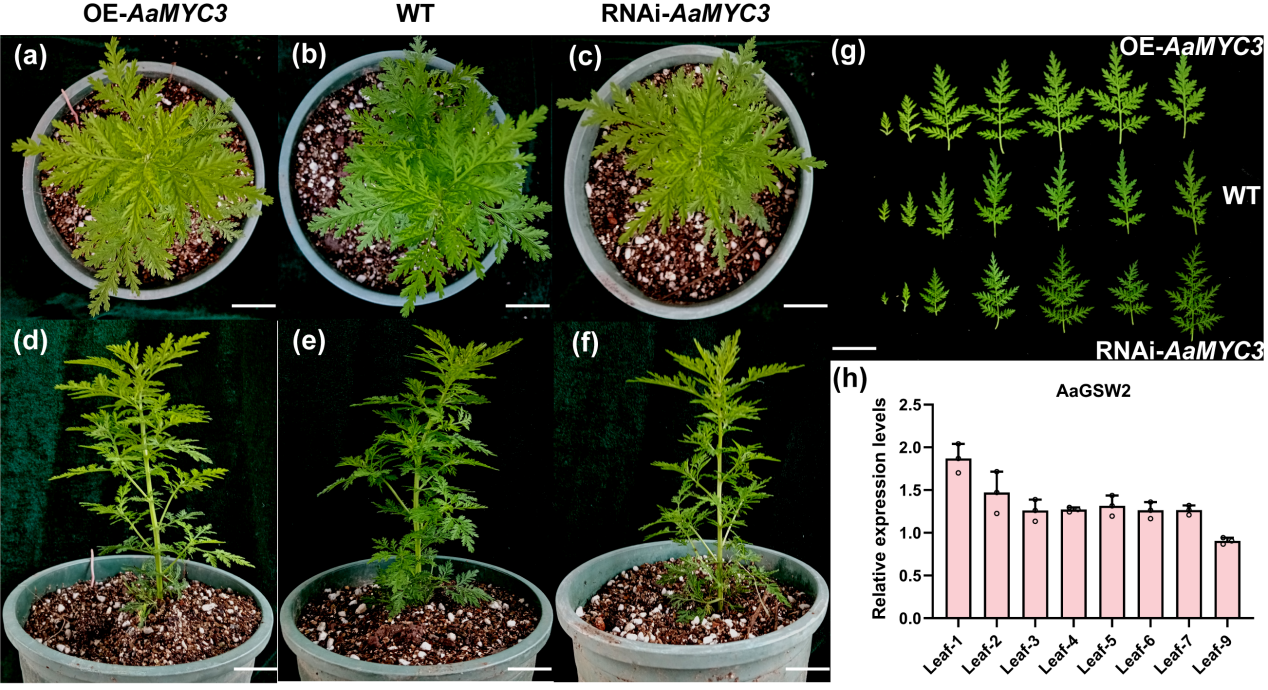
**

**Figure S7** Transgenic *Artemisia annua* leaf phenotype and leaf sequence expression pattern of *AaGSW2*. (a-f) Transgenic *A. annua* plant phenotype. (g) Transgenic *A. annua* leaf phenotype. (h) Relative expression levels of *AaGSW2* in wild-type *A. annua* plants various leaf sequence. All data represent the means ± SDs (n = 3). Bar: 3.5 cm.

**
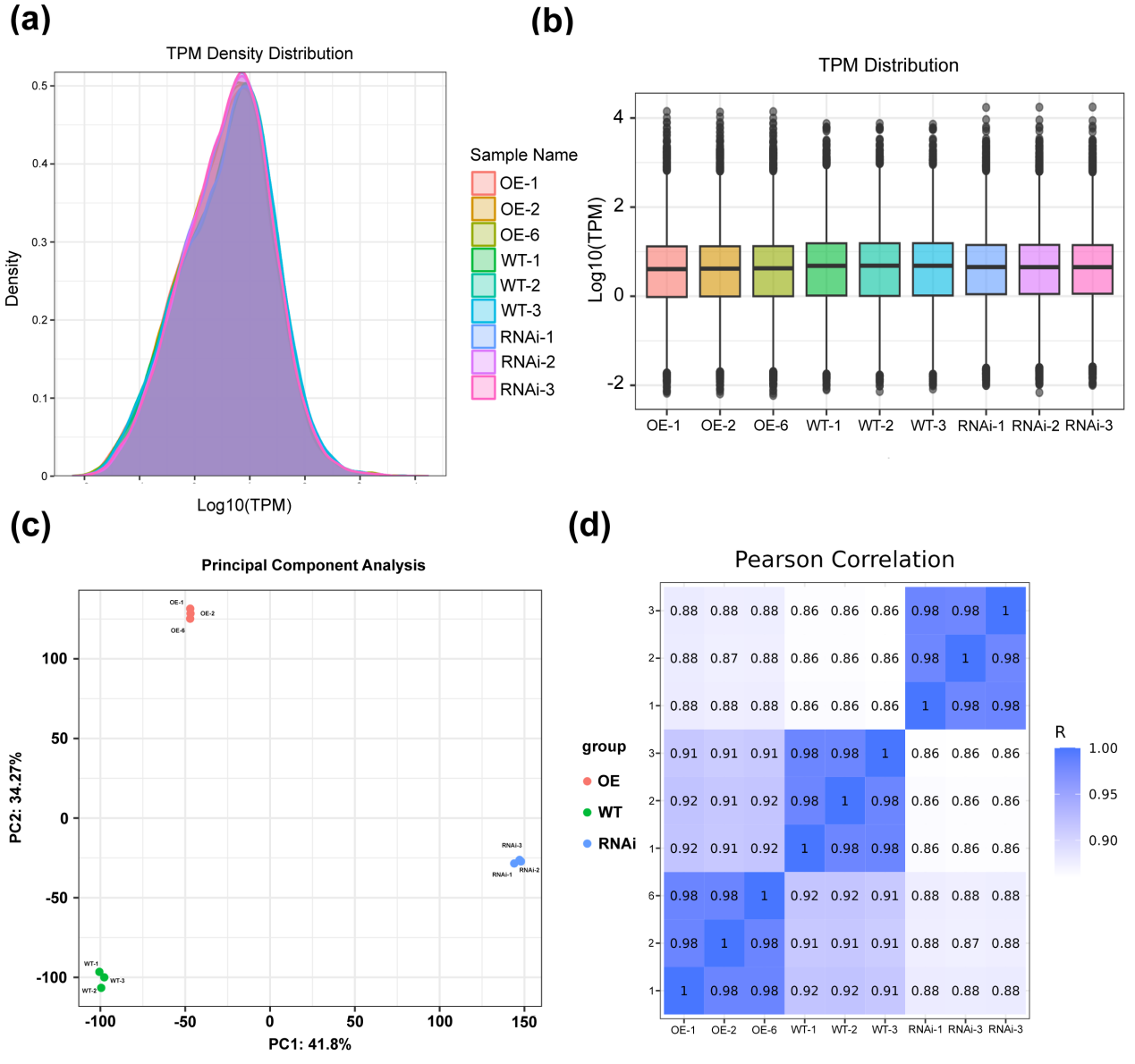
**

**Figure S8** Gene expression abundance statistics of OE-*AaMYC3*, RNAi-*AaMYC3* and wild-type *Artemisia annua* leaves. (a) After calculating all the Transcripts Per kilobase of the exon model per Million mapped reads (TPMs) of each sample, the distribution of gene expression levels of different samples was shown by the TPM density distribution graph. (b) Box graphs showing the distribution of gene expression levels in different samples. (c) Principal Component Analysis (PAC) of all samples. (d) Pearson Correlation checks between different samples.

**
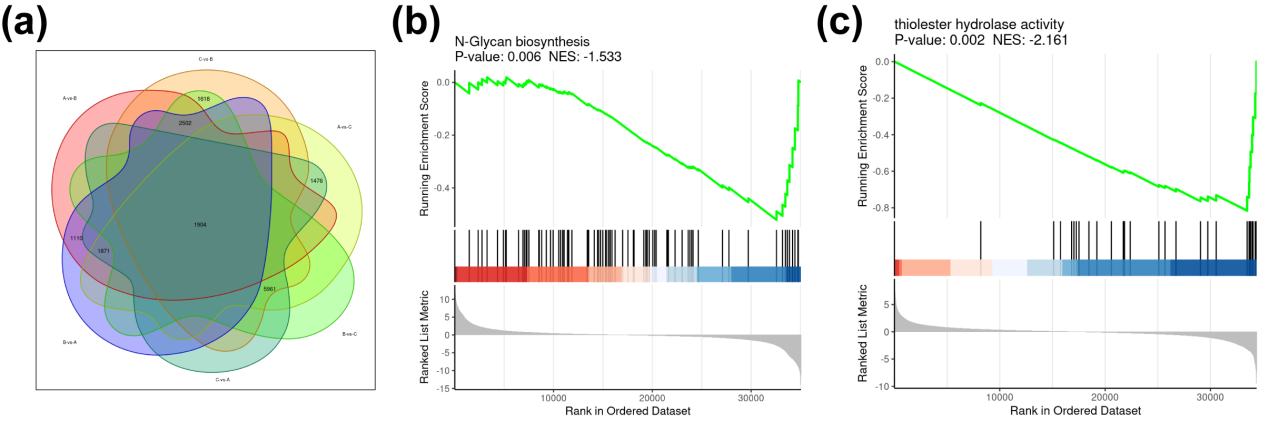
**

**Figure S9** Differential gene analysis and Gene Set Enrichment Analysis (GSEA) of OE-*AaMYC3* (A), RNAi-*AaMYC3* (C) and wild-type (B) *Artemisia annua* leaves genes. (a) Venn graphs of all genes significantly differentially expressed in A, B and C. (b-c) Sesquiterpene metabolism pathway Enrichment Score (ES) folding plot for WT-vs-OE (b) and RNAi-vs-WT (c) in GSEA.


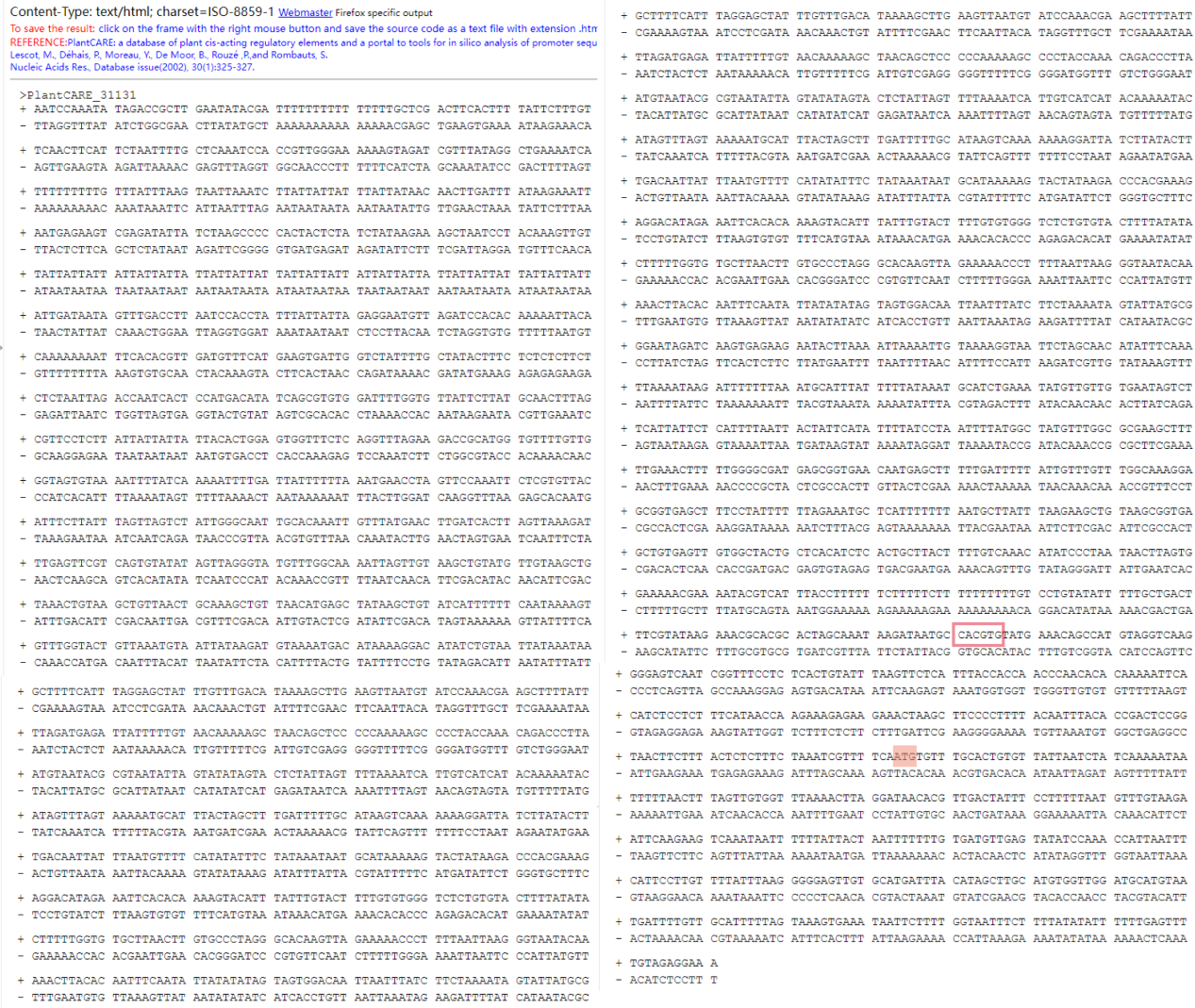


**Figure S10** Nucleotide sequences of *AaHD1* promoters. The red bordered box indicates the G-box sequence and position in the yeast single heterozygote experiment, and the red full box indicates the position of the translation initiation site (ATG).

**
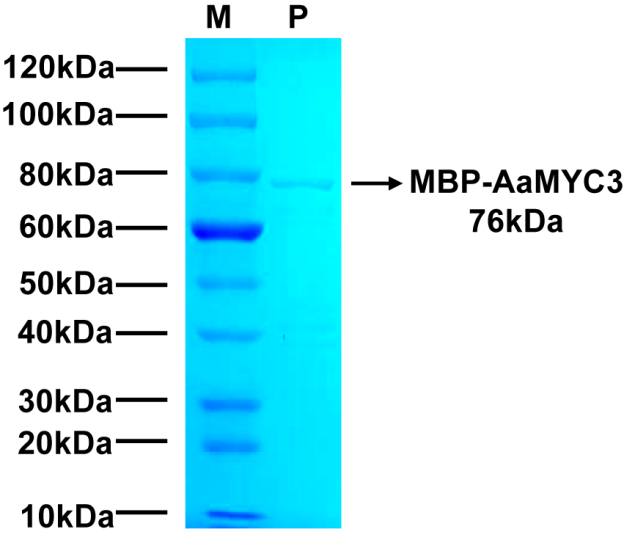
**

**Figure S11** Acquisition of the recombinant protein MBP-AaMYC3. M: Marker, P: SDS-PAGE electrophoregrams of MBP-AaMYC3.


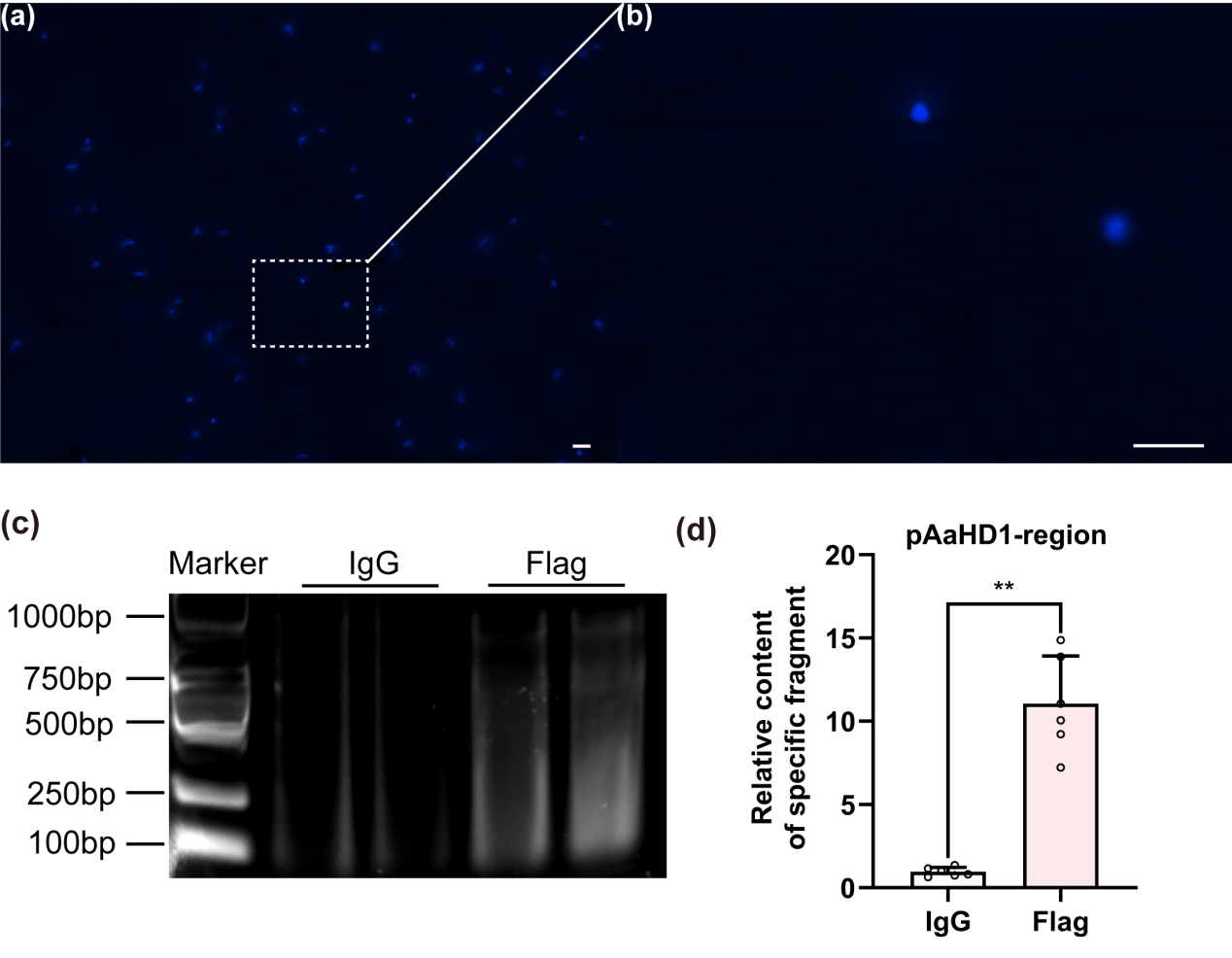


**Figure S12** CUT&tag-qPCR identification of *AaHD1* directly regulated by AaMYC3 in OE-*AaMYC3*-Flag transgenic *Artemisia annua*. (a) DAPI staining of intact nuclei isolated from *A. annua* leaves, 5× objective lens, Bar: 20μm. (b) DAPI (4',6-diamidino-2-phenylindole) staining of intact nuclei isolated from *A. annua* leaves, 10× objective lens, Bar: 20μm. (c) Agarose gel electrophoresis of 2 µL of PCR product from the Cut&tag library. (d) RT-qPCR results show the relative content of fragments from pAaHD1, pCYP71AV1 and pALDH1 in the AaMYC3-Flag assay group compared with the IgG control group. The IgG was normalized to 1. All data represent the mean ± SD (n = 6 independent experiments; **, *P* < 0.01; Student’s *t*-test).

**
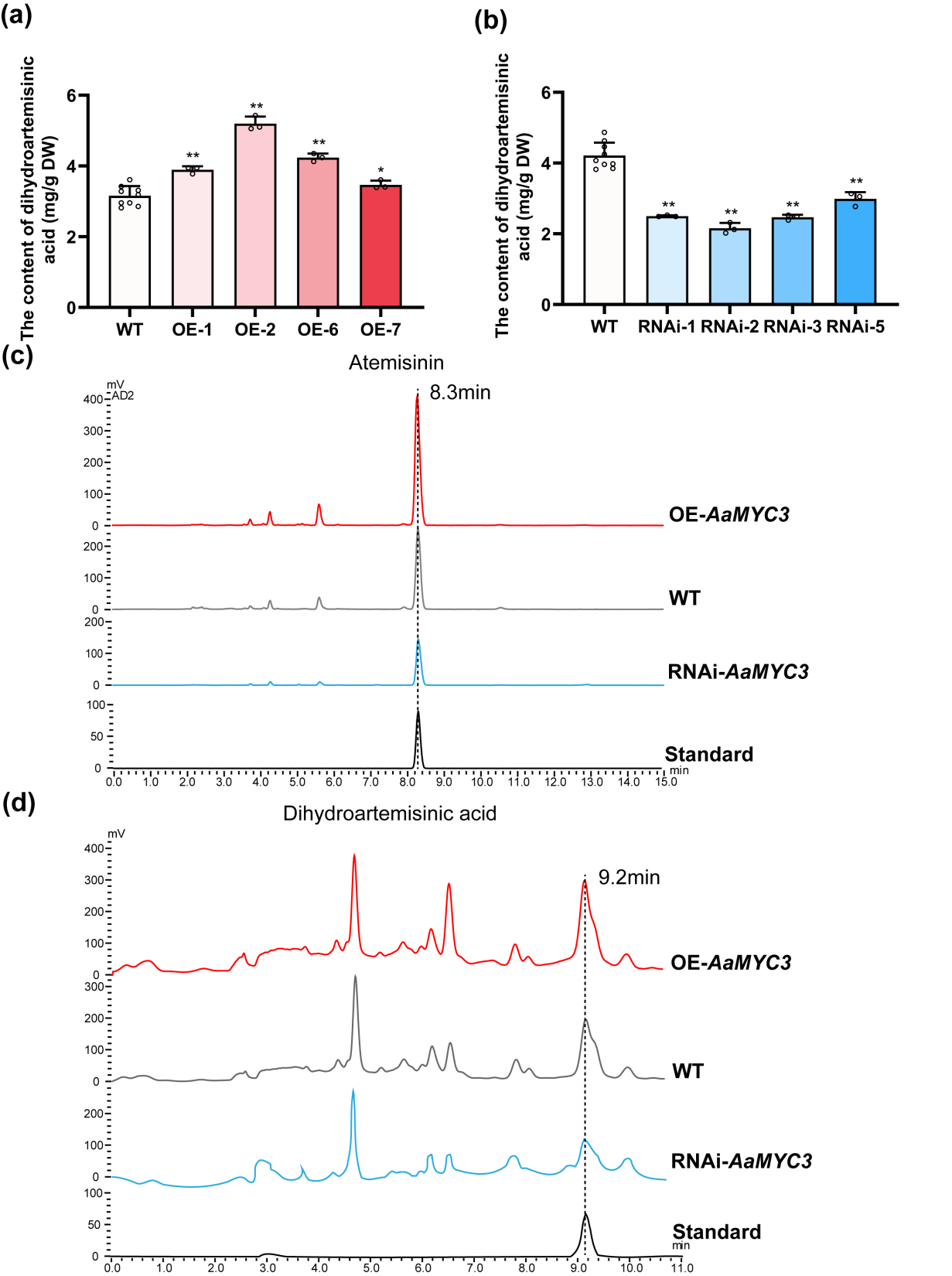
**

**Figure S13** Contents of dihydroartemisinic acid and HPLC chromatograms in transgenic *Artemisia annua* plants. (a) OE-*AaMYC3* lines. (b) RNAi-*AaMYC3* lines. All data represent the mean ± SD (n ≥ 3 independent experiments; *, *P* < 0.05; **, *P* < 0.01; Student’s *t*-test). (c) HPLC chromatograms of artemisinin in transgenic *A. annua* and wild-type. (d) HPLC chromatograms of dihydroartemisinic acid in transgenic *A. annua* and wild-type.


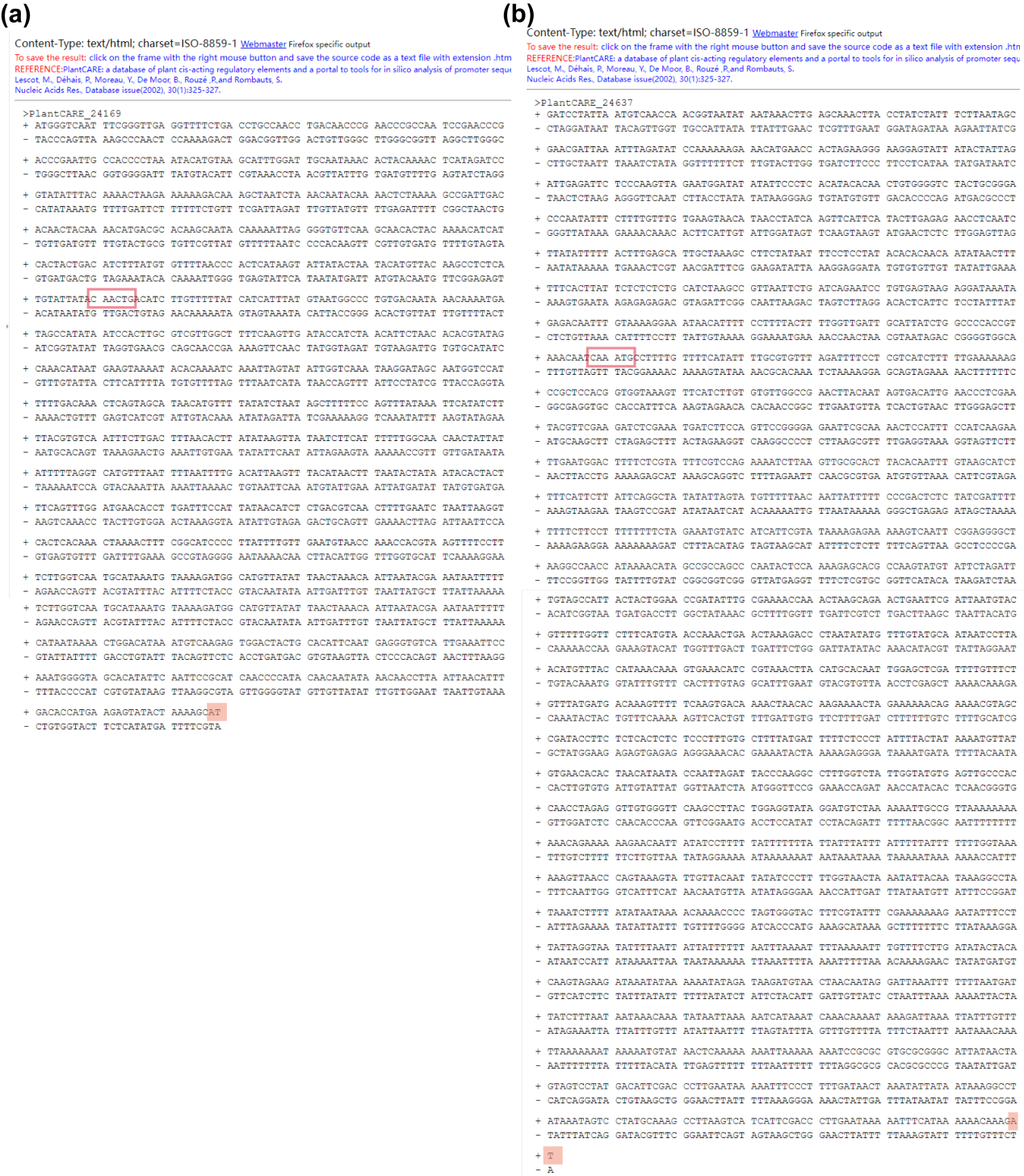


**Figure S14** Nucleotide sequences of *CYP71AV1* and *ALDH1* promoters. (a) *CYP71AV1* promoter. (b) *ALDH1* promoter. The red bordered box indicates the E-box sequence and position in the yeast single heterozygote experiment, and the red full box indicates the position of the translation initiation site (ATG).

**
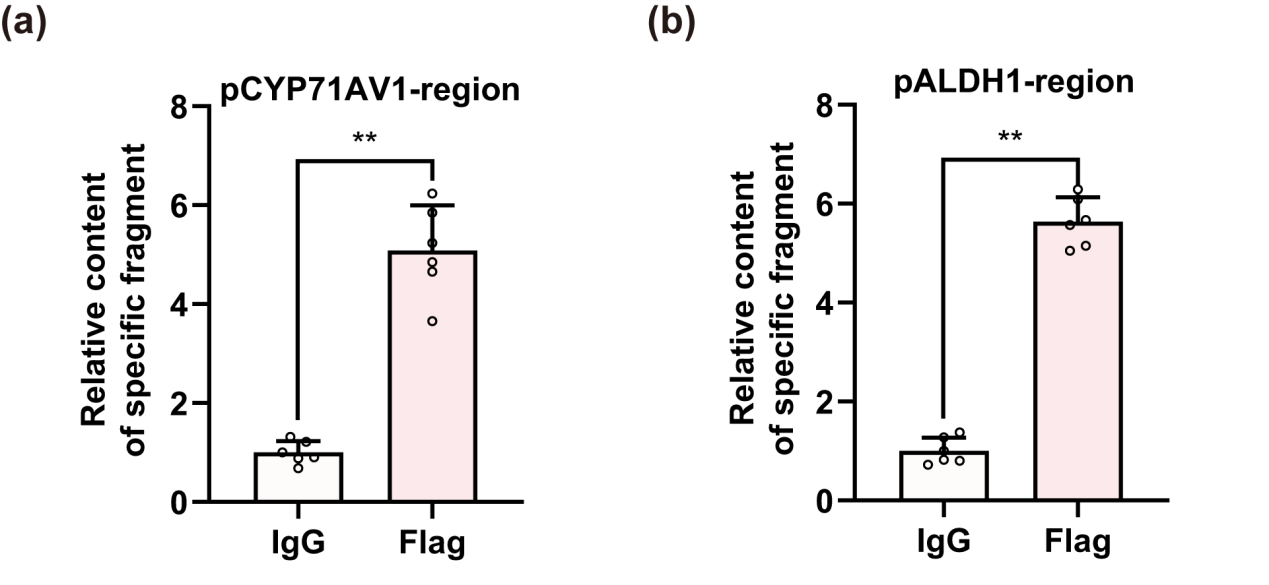
**

**Figure S15** CUT&tag-qPCR identification of *CYP71AV1* (a) and *ALDH1* (b) directly regulated by AaMYC3 in OE-*AaMYC3*-Flag transgenic *Artemisia annua*. The IgG was normalized to 1. All data represent the mean ± SD (n = 6 independent experiments; **, *P* < 0.01; Student’s *t*-test).

**
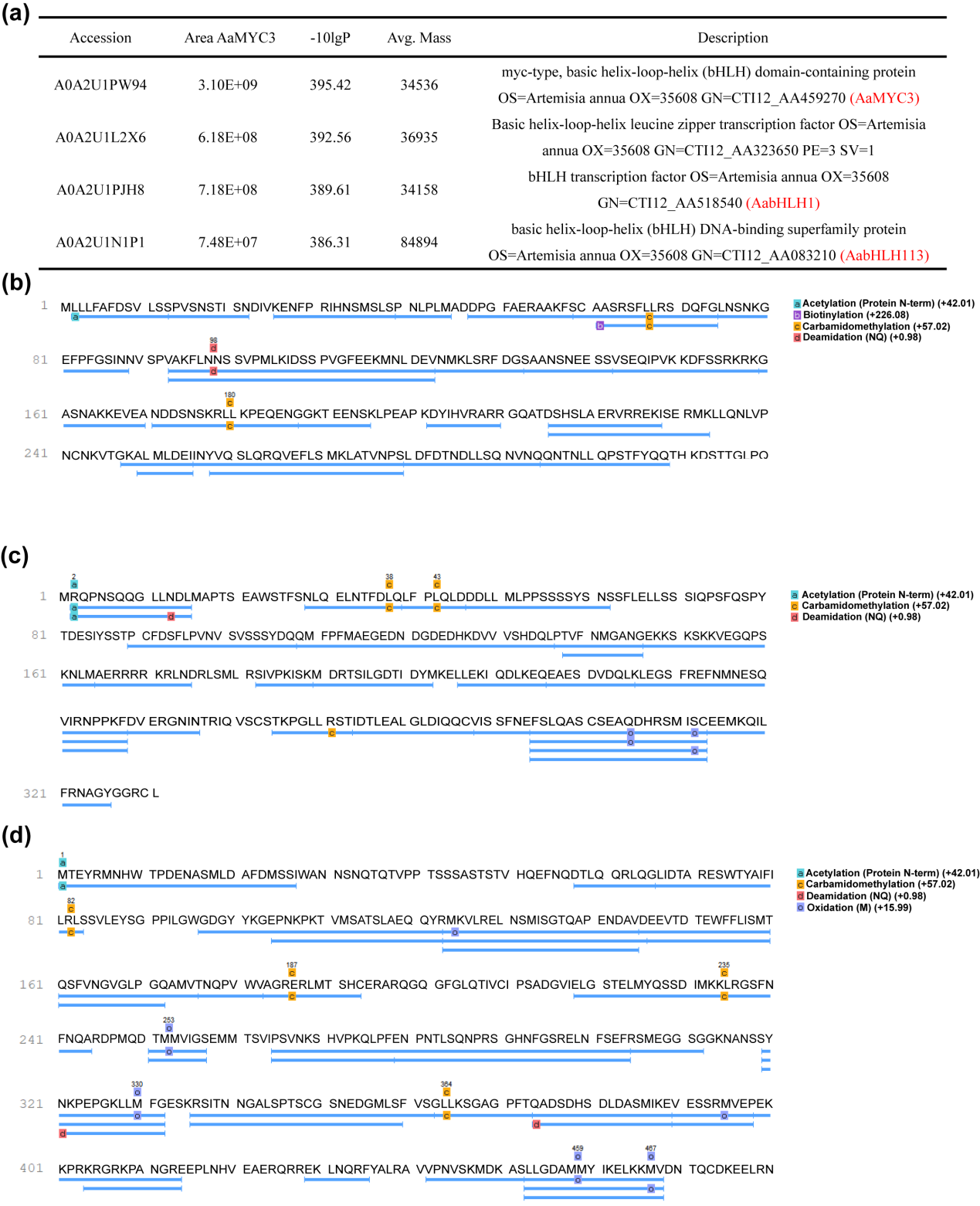
**

**Figure S16** IP-MS screening of proteins interacting with AaMYC3 in OE-*AaMYC3* transgenic *Artemisia annua*. (a) Proteins with the highest abundance sequenced by IP-MS. (b-d) The sequenced peptides were sequence aligned with the *A. annua* proteome and screened for AabHLH113 (b), AaMYC3 (c) and AabHLH1 (d).

**
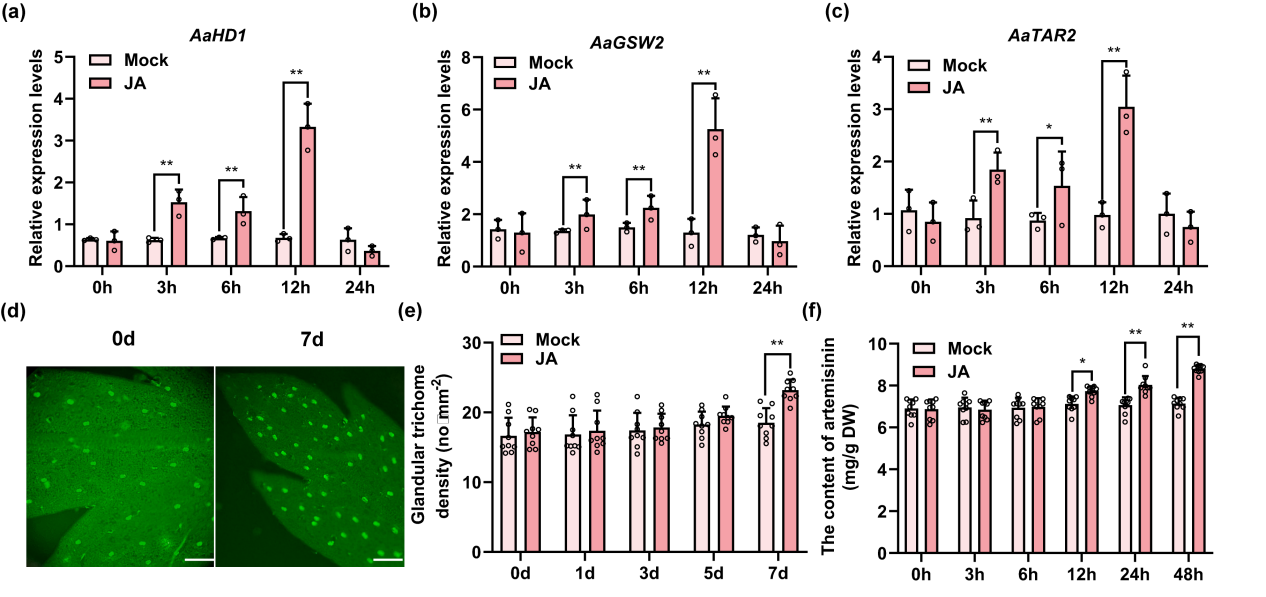
**

**Figure S17** JA induced the content of GST density and artemisinin as well as the expression levels of *AaHD1*, *AaGSW2*, *AaTAR2* and artemisinin biosynthetic genes in *Artemisia annua*. (a-c) Relative expression levels of *AaHD1* (a), *AaGSW2* (b), and *AaTAR2* (c) after methyl jasmonate (MeJA) treatment. All data represent the means ± SDs (n = 3 as indicated in the figure; *, *P* < 0.05; **, *P* < 0.01; Student’s *t*-test). (d) Imaging of glandular trichomes on the adaxial side of mature leaves of wild-type *A. annua* plants after methyl jasmonate (MeJA) treatment. Green fluorescent glandular trichomes were captured by the yellow autofluorescence of the glandular trichomes and the blue autofluorescence of the Chl fluorescence microscope. Bars: 200 μm. (e) Density of glandular trichomes on mature leaves the adaxial side of wild-type *A. annua* plants after methyl jasmonate (MeJA) treatment. (f) Contents of artemisinin in wild-type *A. annua* plants after methyl jasmonate (MeJA) treatment. All data represent the means ± SDs (n = 9 as indicated in the figure; *, *P* < 0.05; **, *P* < 0.01; Student’s *t*-test).

**Table S1** IP-MS screening of 24 proteins interacting with AabHLH113.

| Accession | Area AabHLH113 | Avg. Mass | Description |
| --- | --- | --- | --- |
| A0A2U1MVA7 | 3.04E+09 | 112760 | basic helix-loop-helix (bHLH) DNA-binding superfamily protein OS=Artemisia annua OX=35608 GN=CTI12_AA083210 PE=4 SV=1 |
| A0A2U1PJW5 | 8.60E+10 | 262090 | myc-type, basic helix-loop-helix (bHLH) domain-containing protein OS=Artemisia annua OX=35608 GN=CTI12_AA459270 PE=3 SV=1 |
| A0A2U1PD29 | 8.16E+10 | 314350 | ZIM-domain protein 8 OS=Artemisia annua OX=35608 GN=CTI12_AA166590 PE=3 SV=1 |
| A0A2U1LDK5 | 1.67E+09 | 200931 | Basic helix-loop-helix leucine zipper transcription factor OS=Artemisia annua OX=35608 GN=CTI12_AA323650 PE=3 SV=1 |
| A0A2U1Q435 | 4.96E+09 | 366720 | bHLH transcription factor OS=Artemisia annua OX=35608 GN=CTI12_AA290070 PE=3 SV=1 |
| A0A2U1N4R4 | 8.05E+06 | 79934 | Dirigent protein OS=Artemisia annua OX=35608 GN=CTI12_AA061840 PE=3 SV=1 |
| A0A2U1QF82 | 3.94E+06 | 79935 | DNA damage-binding protein 1 OS=Artemisia annua OX=35608 GN=CTI12_AA038430 PE=3 SV=1 |
| A0A2U1LFR8 | 9.57E+06 | 92736 | NAD(P)-binding Rossmann-fold superfamily protein OS=Artemisia annua OX=35608 GN=CTI12_AA495850 PE=4 SV=1 |
| A0A2U1QGW5 | 1.59E+06 | 90620 | HOPM interactor 7 OS=Artemisia annua OX=35608 GN=CTI12_AA030790 PE=4 SV=1 |
| A0A2U1QAE6 | 2.00E+06 | 26918 | interacting MYB domain-containing protein OS=Artemisia annua OX=35608 GN=CTI12_AA054940 PE=4 SV=1 |
| A0A2U1LPF1 | 5.06E+06 | 73250 | interacting MYB domain-containing protein OS=Artemisia annua OX=35608 GN=CTI12_AA468870 PE=3 SV=1 |
| A0A2U1QGE3 | 5.06E+06 | 44182 | interacting MYB domain-containing protein OS=Artemisia annua OX=35608 GN=CTI12_AA032840 PE=3 SV=1 |
| A0A2U1KJZ2 | 5.06E+06 | 52127 | interacting MYB domain-containing protein OS=Artemisia annua OX=35608 GN=CTI12_AA593750 PE=3 SV=1 |
| A0A2U1L4X5 | 5.06E+06 | 52932 | interacting MYB domain-containing protein OS=Artemisia annua OX=35608 GN=CTI12_AA530840 PE=3 SV=1 |
| A0A2U1MCF3 | 1.31E+06 | 14648 | Basic helix-loop-helix leucine zipper transcription factor OS=Artemisia annua OX=35608 GN=CTI12_AA395750 PE=3 SV=1 |
| A0A2U1MF21 | 1.31E+06 | 14682 | Basic helix-loop-helix leucine zipper transcription factor OS=Artemisia annua OX=35608 GN=CTI12_AA386040 PE=3 SV=1 |
| A0A2U1MRE2 | 1.90E+06 | 16122 | Basic helix-loop-helix leucine zipper transcription factor OS=Artemisia annua OX=35608 GN=CTI12_AA351190 PE=4 SV=1 |
| A0A2U1L3T2 | 4.04E+06 | 27208 | NAD(P)-binding domain-containing protein OS=Artemisia annua OX=35608 GN=CTI12_AA529860 PE=4 SV=1 |
| A0A2U1LL23 | 4.04E+06 | 34145 | NAD(P)-binding domain-containing protein OS=Artemisia annua OX=35608 GN=CTI12_AA480230 PE=4 SV=1 |
| A0A2U1KZN4 | 9.64E+06 | 17825 | Basic helix-loop-helix leucine zipper transcription factor OS=Artemisia annua OX=35608 GN=CTI12_AA544680 PE=4 SV=1 |
| A0A2U1QJA8 | 1.83E+06 | 71075 | Basic helix-loop-helix leucine zipper transcription factor OS=Artemisia annua OX=35608 GN=CTI12_AA022900 PE=3 SV=1 |
| A0A2U1M510 | 1.30E+07 | 36473 | Basic helix-loop-helix leucine zipper transcription factore OS=Artemisia annua OX=35608 GN=CTI12_AA419400 PE=3 SV=1 |
| A0A2U1KZS6 | 6.20E+06 | 79159 | NADPH--cytochrome P450 reductase 2 OS=Artemisia annua OX=35608 GN=CPR2 PE=3 SV=1 |
| A0A2U1NAP8 | 6.20E+06 | 78375 | NADPH--cytochrome P450 reductase OS=Artemisia annua OX=35608 GN=CTI12_AA286710 PE=3 SV=1 |

**Table S2** IP-MS screening of 21 proteins interacting with AaMYC3.

| Accession | Area AaMYC3 | Avg. Mass | Description |
| --- | --- | --- | --- |
| A0A2U1PW94 | 3.10E+09 | 34536 | myc-type, basic helix-loop-helix (bHLH) domain-containing protein OS=Artemisia annua OX=35608 GN=CTI12_AA459270 PE=3 SV=1 |
| A0A2U1L2X6 | 6.18E+08 | 36935 | Basic helix-loop-helix leucine zipper transcription factor OS=Artemisia annua OX=35608 GN=CTI12_AA323650 PE=3 SV=1 |
| A0A2U1PJH8 | 7.18E+08 | 34158 | bHLH transcription factor OS=Artemisia annua OX=35608 GN=CTI12_AA518540 PE=3 SV=1 |
| A0A2U1N1P1 | 7.48E+07 | 84894 | basic helix-loop-helix (bHLH) DNA-binding superfamily protein OS=Artemisia annua OX=35608 GN=CTI12_AA083210 PE=4 SV=1 |
| A0A2U1N9G2 | 4.51E+06 | 75345 | bHLH transcription factor OS=Artemisia annua OX=35608 GN=CTI12_AA291740 PE=4 SV=1 |
| A0A2U1KGA4 | 2.95E+07 | 71736 | NAC domain containing protein OS=Artemisia annua OX=35608 GN=CTI12_AA605910 PE=3 SV=1 |
| A0A2U1Q583 | 1.95E+07 | 81147 | NAC domain containing protein OS=Artemisia annua OX=35608 GN=CTI12_AA440100 PE=3 SV=1 |
| A0A2U1P9A7 | 1.10E+06 | 26918 | basic helix-loop-helix (bHLH) domain-containing protein OS=Artemisia annua OX=35608 GN=CTI12_AA054940 PE=4 SV=1 |
| A0A2U1KXF9 | 1.18E+06 | 50911 | basic helix-loop-helix (bHLH) domain-containing protein OS=Artemisia annua OX=35608 GN=CTI12_AA554270 PE=3 SV=1 |
| A0A2U1L0A5 | 1.18E+06 | 46133 | basic helix-loop-helix (bHLH) domain-containing protein OS=Artemisia annua OX=35608 GN=CTI12_AA544540 PE=3 SV=1 |
| A0A2U1MQF4 | N/A | 14716 | basic helix-loop-helix (bHLH) domain-containing protein OS=Artemisia annua OX=35608 GN=CTI12_AA311710 PE=3 SV=1 |
| A0A2U1PGK2 | N/A | 18794 | NAD(P)-binding Rossmann OS=Artemisia annua OX=35608 GN=CTI12_AA099120 PE=3 SV=1 |
| A0A2U1QDJ6 | 3.23E+07 | 45617 | NAD(P)-binding Rossmann OS=Artemisia annua OX=35608 GN=CTI12_AA039190 PE=3 SV=1 |
| A0A2U1MS44 | 5.32E+06 | 52667 | NAD(P)-binding Rossmann OS=Artemisia annua OX=35608 GN=CTI12_AA347600 PE=4 SV=1 |
| A0A2U1NLV7 | 8.12E+05 | 63940 | Basic helix-loop-helix leucine zipper transcription factor OS=Artemisia annua OX=35608 GN=CTI12_AA249410 PE=3 SV=1 |
| A0A2U1P1P5 | 8.12E+05 | 82844 | NAC domain containing protein OS=Artemisia annua OX=35608 GN=CTI12_AA204730 PE=3 SV=1 |
| A0A2U1L5L6 | 4.65E+06 | 93448 | Basic helix-loop-helix leucine zipper transcription factor OS=Artemisia annua OX=35608 GN=CTI12_AA528380 PE=4 SV=1 |
| A0A2U1KR85 | 4.65E+06 | 25612 | Basic helix-loop-helix leucine zipper transcription factor OS=Artemisia annua OX=35608 GN=CTI12_AA572930 PE=4 SV=1 |
| A0A2U1N9E7 | 4.65E+06 | 59398 | Basic helix-loop-helix leucine zipper transcription factor OS=Artemisia annua OX=35608 GN=CTI12_AA291430 PE=3 SV=1 |
| A0A2U1NYE7 | 4.65E+06 | 59097 | Basic helix-loop-helix leucine zipper transcription factor OS=Artemisia annua OX=35608 GN=CTI12_AA214450 PE=3 SV=1 |
| A0A2U1KIB9 | 4.65E+06 | 41328 | Basic helix-loop-helix leucine zipper transcription factor OS=Artemisia annua OX=35608 GN=CTI12_AA599490 PE=4 SV=1 |

**N/A: Not Available**

**Table S3** Sequences of primers used in molecular assays.

| Primer names | sequences | Application |
| --- | --- | --- |
| AaMYC3-F | GCGCATCAAACGCGAAAAAG | qRT-PCR |
| AaMYC3-R | AATCCTTTGGTGCTTCGGGT | qRT-PCR |
| ADS-F | GGGAGATCAGTTTCTCATCTATGAA | qRT-PCR |
| ADS-R | CTTTTAGTAGTTGCCGCACTTCTT | qRT-PCR |
| CYP71AV1-F | ACTGACCACTTCCATTGCTCTTG | qRT-PCR |
| CYP71AV1-R | ACTTTCTGGCTAAATCCCTAACCC | qRT-PCR |
| DBR2-F | ATCATCAACAAGCAAGCCCATTTC | qRT-PCR |
| DBR2-R | GCGATAGTCTTCAACCACCTCTAG | qRT-PCR |
| ALDH1-F | CGGAGTAGTTGGTCACATCATTC | qRT-PCR |
| ALDH1-R | TTAATCACGCCATCAGGAACAC | qRT-PCR |
| *β*-actin-F | CCAGGCTGTTCAGTCTCTGTAT | qRT-PCR |
| *β*-actin-R | CGCTCGGTAAGGATCTTCATCA | qRT-PCR |
| AaHD1-F | TCCGAGAATGGAAGTTGGGC | qRT-PCR |
| AaHD1-R | GTGAAAGCTGTCGAGTGGGA | qRT-PCR |
| AaGSW2-F | TTCCTCATCTTCAACTTCACCAC | qRT-PCR |
| AaGSW2-R | CGCTACTCGTGGGAGAAACAT | qRT-PCR |
| AaTAR2-F | CCACCGGTTTCTGCATTTTC | qRT-PCR |
| AaTAR2-R | TGTCCTCGTTGCACAAGAAG | qRT-PCR |
| pADS-F | gcctcgagTATGGTGTTTCAACGCTTAT | Dual-luc |
| pADS-R | gcggatccACTAGAGTTGCTCTTAGC | Dual-luc |
| pCYP71AV1-F | gcctcgagTTTTCTGACCTGCCAACC | Dual-luc |
| pCYP71AV1-R | gcggatccCATGCTTTTAGTATACTC | Dual-luc |
| pDBR2-F | gcctcgagCCCCAATGGGTTGGTCTA | Dual-luc |
| pDBR2-R | gcggatccTAAACAGCCCTTGTTTTGCA | Dual-luc |
| pALDH1-F | gcctcgagAAGGGAAGGAGTATTATACT | Dual-luc |
| pALDH1-R | gcggatccAAAAGCACAAAGGGAGAGA | Dual-luc |
| pAaHD1-F | gcctcgagCACAATTTCAATATTATATA | Dual-luc |
| pAaHD1-R | gcggatccGTGGTAAATGAGAACTTAAA | Dual-luc |
| AaMYC3-F | ttcctgcagcccgggggatccATGGAGCTTCTATTC | Overexpression  Plasmited/Dual-luc |
| AaMYC3-R | cgcggtggcggccgctctagaTTGGGGCAGACCCGT | Overexpression  Plasmited/Dual-luc |
| AabHLH1-F | ttcctgcagcccgggggatccATGACGGAGTACCGCA | Overexpression  Plasmited/Dual-luc |
| AabHLH1-R | cgcggtggcggccgctctagaAATGGATCGGAAAATC | Overexpression  Plasmited/Dual-luc |
| AabHLH1113-F | ttcctgcagcccgggggatccATGCACCAACCAAAC | Overexpression  Plasmited/Dual-luc |
| AabHLH113-R | cgcggtggcggccgctctagaTTACAAGCATCTGCC | Overexpression  Plasmited/Dual-luc |
| AaMYC3-F | cacagttcgagaagctcgagATGGAGCTTCTATTC | BiLC |
| AaMYC3-R | agatctggtcgactactcgagTTGGGGCAGACCCGT | BiLC |
| AabHLH1-F | ccacagttcgagaagctcgagATGACGGAGTACCGCA | BiLC |
| AabHLH1-R | tctgcaggtcgactactcgagAATGGATCGGAAAATC | BiLC |
| AabHLH1113-F | ccacagttcgagaagctcgagATGCACCAACCAAAC | BiLC |
| AabHLH113-R | tctgcaggtcgactactcgagTTACAAGCATCTGCC | BiLC |
| AaMYC3-RNAi-F | gcggtaccaagcttACCCGAAGCACCAAAGGATT | RNAi plasmited |
| AaMYC3-RNAi-R | gctctagactcgagGCAGACCCGTTGTAGAGTCC | RNAi plasmited |
| 35S-F | tgtgatatctccactgacgtaagggat | Transganic plants PCR |
| rbcs-R | cacacaccagaatcctactga | Transganic plants PCR |
| OCS-R | GGATCTGAGCTACACATGCT | Transganic plants PCR |
| Hygr-F | ATGAAAAAGCCTGAACTCAC | Overexpression  plasmited |
| Hygr-R | TTTCTTTGCCCTCGGACGAG | Overexpression  plasmited |
| Kan-F | AATCCATCTTGTTCAATCAT | RNAi plasmited |
| Kan-R | TTCTTGACGAGTTCTTCTGA | RNAi plasmited |
| AaMYC3-c5X-F | gcggatccATGGAGCTTCTATTC | Protein expression plasmited |
| AaMYC3-c5X-R | gcctgcagTTGGGGCAGACCCGT | Protein expression plasmited |
| AabHLH1-32a-F | gcggatccAATGGATCGGAAAATC | Protein expression plasmited |
| AabHLH1-32a-R | gcctcgagAATGGATCGGAAAATC | Protein expression plasmited |
| AabHLH113-c5X-F | gcggatccATGCACCAACCAAAC | Protein expression plasmited |
| AabHLH113-c5X-R | gcctgcagTTACAAGCATCTGCC | Protein expression plasmited |
| AaMYC3-1300-F | gcggatccATGGAGCTTCTATTC | GFP fusion expression plasmited |
| AaMYC3-1300-R | gctctagaTTGGGGCAGACCCGT | GFP fusion expression plasmited |
| pAaMYC3-1305.1-F | gcgaattcTAAAGTACTTATAAAAAAAA | Promote GUS expression plasmited |
| pAaMYC3-1305.1-R | gccaatggCTGTTGGTCGAACCTTTTAG | Promote GUS expression plasmited |
| N501-index 2 (i5) | TAGATCGC | CUT&Tag |
| N701-index 1 (i7) | TAAGGCGA | CUT&Tag |
| pCYP71AV1-region-F | TAATACATGTTACAAGCCTC | CUT&Tag-qPCR |
| pCYP71AV1-region-R | GACCAATATACTAATTTGAT | CUT&Tag-qPCR |
| pALDH1-region-F | TCAGAATCCTGTGAGTAAGA | CUT&Tag-qPCR |
| pALDH1-region-R | GGCCAACACACAAGATGAAA | CUT&Tag-qPCR |
| pAaHD1-region-F | TTTTTTTTTTGTCCTGTATA | CUT&Tag-qPCR |
| pAaHD1-region-R | GTGGTAAATGAGAACTTAAA | CUT&Tag-qPCR |
| *β*-actin-F | AACCATCAGTAGTCTGTTCCA | CUT&Tag-qPCR |
| *β*-actin-R | CCTTGGTAATGAAAACACAAG | CUT&Tag-qPCR |
| RNAi-*AaMYC3* fragment | ACCCGAAGCACCAAAGGATTATATTCATGTTAGAGCAAGAAGAGGACAAGCTACTGATAGTCATAGTTTAGCAGAAAGAGTTAGAAGAGAAAAGATTAGTGAAAGAATGAAGCTTCTTCAGAATCTTGTGCCAAATTGTAACAAGGTAACAGGAAAGGCACTTATGCTTGATGAAATCATCAATTACGTGCAATCGTTGCAAAGACAAGTTGAGTTCCTTTCGATGAAGTTAGCTACGGTGAACCCAAGTCTCGACTTTGACACCAATGACCTACTCTCGCAAAATGTTAATCAACAAAACACAAATCTACTACAACCATCGACTTTTTATCAACAAACACACAAGGACTCTACAACGGGTCTGC | RNAi plasmid |

**Table S4** Sequences of primers used in yeast one-hybrid assays.

| Primer names | Sequences (5’-3’) |
| --- | --- |
| pADS-E1-F | gcggtaccTATGGTGTTTCAACGCTTAT |
| pADS-E1-R | gcctcgagAATGTGTTAGGATAGTGTAA |
| pADS-E2-F | gcggtaccTATCACTTCTTTTAATGCTG |
| pADS-E2-R | gcctcgagACTAGAGTTGCTCTTAGC |
| pDBR2-E1-F | gcggtaccTGGCCGACGGCACCTGAA |
| pDBR2-E-R | gcctcgagGATATAATGCCAGCGCAC |
| pDBR2-E2-F | gcggtaccCATTTCGTTTATCAATTTTG |
| pDBR2-E2-R | gcctcgagATGACAAGTATAGTGCTTTA |
| pCYP71AV1-Ebox-F | cCAAGCCTCTCATGTATTATACAACTGACATCTTGTTTTTATCATCA |
| pCYP71AV1-Ebox-R | tcgagTGATGATAAAAACAAGATGTCAGTTGTATAATACATGAGAGGCTTG |
| pCYP71AV1-mEbox-F | cCAAGCCTCTCATGTATTATACTTTTGACATCTTGTTTTTATCATCA |
| pCYP71AV1-mEbox-R | tcgagTGATGATAAAAACAAGATGTCAAAAGTATAATACATGAGAGGCTTG |
| pALDH1-Ebox-F | cTCAGAATCCTGTGAGTAAGAGGATAAATAGAGACAATTTGTAAAAGGAAATAACATTTTCCTTTTACTTTTGGTTGATTGCATTATCTGGCCCCACCGTAAACAATCAAATGCCTTTTGTTTTCATATTTTG |
| pALDH1-Ebox-R | tcgagGGCCAACACACAAGATGAAACTTTACCACCGTGGAGCGGCTTTTTTCAAAAAGATGACGAGGAAAATCTAAACACGCAAAATATGAAAACAAAAGGCATTTGATTGTTTACGGTGGGGCCAG |
| pALDH1-mEbox-F | cTCAGAATCCTGTGAGTAAGAGGATAAATAGAGACAATTTGTAAAAGGAAATAACATTTTCCTTTTACTTTTGGTTGATTGCATTATCTGGCCCCACCGTAAACAATCTTTTGCCTTTTGTTTTCATATTTTG |
| pALDH1-mEbox-R | tcgagGGCCAACACACAAGATGAAACTTTACCACCGTGGAGCGGCTTTTTTCAAAAAGATGACGAGGAAAATCTAAACACGCAAAATATGAAAACAAAAGGCAAAAGATTGTTTACGGTGGGGCCAG |
| pAaHD1-Gbox-F | cAGCAAATAAGATAATGCCACGTGTATGAAACAGCCATGTAGGTCAA |
| pAaHD1-Gbox-R | tcgagTTGACCTACATGGCTGTTTCATACACGTGGCATTATCTTATTTGCT |
| pAaHD1-mGbox-F | cAGCAAATAAGATAATGCAACGTGTATGAAACAGCCATGTAGGTCAA |
| pAaHD1-mGbox-R | tcgagTTGACCTACATGGCTGTTTCATACACGTTGCATTATCTTATTTGCT |
| AaMYC3-42AD-F | gcctctcccATGGAGCTTCTATTC |
| AaMYC3-42AD-R | ccaaagcttTTGGGGCAGACCCGT |

**Table S5** Sequences of primers used in yeast two-hybrid assays.

| Probe names | Sequences (5’-3’) |
| --- | --- |
| AaMYC3-F | GCCATGGAGGCCAGTGAATTCATGGAGCTTCTATTC |
| AaMYC3-R | ATGCCCACCCGGGTGGAATCCTTGGGGCAGACCCGT |
| AabHLH1-F | AGGCCGAATTCCCGGGGATCCATGACGGAGTACCGCA |
| AabHLH1-R | CCGCTGCAGGTCGACGAATCCAATGGATCGGAAAATC |
| AabHLH1113-F | AGGCCGAATTCCCGGGGATCCATGCACCAACCAAAC |
| AabHLH113-R | CCGCTGCAGGTCGACGAATCCTTACAAGCATCTGCC |

**Table S6** Sequences of primers used in Co-immunoprecipitation assays.

| Probe names | Sequences (5’-3’) |
| --- | --- |
| AaMYC3-F | ATACTCGACCCCGGGGGATCCATGGAGCTTCTATTC |
| AaMYC3-R | AGTTCTAGAGTCGACGGATCCTTGGGGCAGACCCGT |
| AabHLH1-F | gagctcggtacccggggatccATGACGGAGTACCGCA |
| AabHLH1-R | caggtcgactctagaggatccAATGGATCGGAAAATC |
| AabHLH1113-F | gagctcggtacccggggatccATGCACCAACCAAAC |
| AabHLH113-R | tcaggtcgactctagaggatccTTACAAGCATCTGCC |

**Table S7** Sequences of probes used in electrophoretic mobility shift assays.

| Probe names | Sequences (5’-3’) |
| --- | --- |
| pCYP71AV1-Ebox-F | CAAGCCTCTCATGTATTATACAACTGACATCTTGTTTTTATCATCA |
| pALDH1-Ebox-F | CTGGCCCCACCGTAAACAATCAAATGCCTTTTGTTTTCATATTTTG |
| pAaHD1-Gbox-F | AGCAAATAAGATAATGCCACGTGTATGAAACAGCCATGTAGGTCAA |
| pCYP71AV1-mEbox-F | CAAGCCTCTCATGTATTATACTTTTGACATCTTGTTTTTATCATCA |
| pALDH1-mEbox-F | CTGGCCCCACCGTAAACAATCTTTTGCCTTTTGTTTTCATATTTTG |
| pAaHD1-mGbox-F | AGCAAATAAGATAATGCCTTTTGTATGAAACAGCCATGTAGGTCAA |
| pADS-3×Ebox-F | AATGCAAATGTTGGGGAGCACGTGTTGGGGAGCACATGGGGA |
| pDBR2-Ebox-F | GTTAGAAATTTTTTTCGTGACATGTGAAATTTTTTTTGACCGCTTT |
| pADS-3×mEbox-F | AATGACAATGTTGGGGAGACAATGTTGGGGAGACAATGGGGA |
| pDBR2-mEbox-F | GTTAGAAATTTTTTTCGTGAACAATGAAATTTTTTTTGACCGCTTT |
